# Transcriptome Analysis of Subcutaneous Adipose Tissue from Severely Obese Patients Highlights Deregulation Profiles in Coding and Non-Coding Oncogenes

**DOI:** 10.3390/ijms22041989

**Published:** 2021-02-17

**Authors:** Federica Rey, Letizia Messa, Cecilia Pandini, Rossella Launi, Bianca Barzaghini, Giancarlo Micheletto, Manuela Teresa Raimondi, Simona Bertoli, Cristina Cereda, Gian Vincenzo Zuccotti, Raffaella Cancello, Stephana Carelli

**Affiliations:** 1 Department of Biomedical and Clinical Sciences “L. Sacco”, School of Medicine, University of Milano, Via Grassi 74, 20157 Milano, Italy; federica.rey@unimi.it (F.R.); rossella.launi@studenti.unimi.it (R.L.); gianvincenzo.zuccotti@unimi.it (G.V.Z.); 2 Pediatric Clinical Research Centre Fondazione “Romeo ed Enrica Invernizzi”, University of Milano, Via G.B. Grassi 74, 20157 Milano, Italy; 3 Department of Chemistry, Materials and Chemical Engineering “Giulio Natta”, Politecnico di Milano, Piazza Leonardo da Vinci 32, 20133 Milano, Italy; letizia.messa@mail.polimi.it (L.M.); bianca.barzaghini@polimi.it (B.B.); manuela.raimondi@polimi.it (M.T.R.); 4 Genomic and Post-Genomic Centre, IRCCS Mondino Foundation, 27100 Pavia, Italy; cecilia.pandini@mondino.it (C.P.); cristina.cereda@mondino.it (C.C.); 5 Department of Pathophysiology and Transplantation, INCO and Department of General Surgery, Istituto Clinico Sant’Ambrogio, University of Milan, Via Francesco Sforza 35, 20122 Milano, Italy; giancarlo.micheletto@unimi.it; 6 Obesity Unit—Laboratory of Nutrition and Obesity Research, Department of Endocrine and Metabolic Diseases, IRCCS Istituto Auxologico Italiano, Via Ariosto 9, 20145 Milano, Italy; simona.bertoli@unimi.it (S.B.); r.cancello@auxologico.it (R.C.); 7 International Center for the Assessment of Nutritional Status (ICANS), Department of Food, Environmental and Nutritional Sciences (DeFENS), University of Milan, Via Celoria 2, 20133 Milano, Italy; 8 Department of Pediatrics, Children’s Hospital “V. Buzzi”, Via Lodovico Castelvetro 32, 20154 Milano, Italy

**Keywords:** obesity, cancer, type 2 diabetes, gender, lncRNAs, transcriptional deregulation, oncogenes

## Abstract

Obesity is a major risk factor for a large number of secondary diseases, including cancer. Specific insights into the role of gender differences and secondary comorbidities, such as type 2 diabetes (T2D) and cancer risk, are yet to be fully identified. The aim of this study is thus to find a correlation between the transcriptional deregulation present in the subcutaneous adipose tissue of obese patients and the oncogenic signature present in multiple cancers, in the presence of T2D, and considering gender differences. The subcutaneous adipose tissue (SAT) of five healthy, normal-weight women, five obese women, five obese women with T2D and five obese men were subjected to RNA-sequencing, leading to the identification of deregulated coding and non-coding RNAs, classified for their oncogenic score. A panel of DE RNAs was validated via Real-Time PCR and oncogene expression levels correlated the oncogenes with anthropometrical parameters, highlighting significant trends. For each analyzed condition, we identified the deregulated pathways associated with cancer, the prediction of possible prognosis for different cancer types and the lncRNAs involved in oncogenic networks and tissues. Our results provided a comprehensive characterization of oncogenesis correlation in SAT, providing specific insights into the possible molecular targets implicated in this process. Indeed, the identification of deregulated oncogenes also in SAT highlights hypothetical targets implicated in the increased oncogenic risk in highly obese subjects. These results could shed light on new molecular targets to be specifically modulated in obesity and highlight which cancers should receive the most attention in terms of better prevention in obesity-affected patients.

## 1. Introduction

Obesity is one of the most serious public health problems worldwide, as, according to the World Health Organization (WHO), obesity has reached epidemic proportions globally, with at least 2.8 million people dying each year as a result of being overweight or obese [1,2]. Obesity is a major risk factor for a number of chronic diseases, including cardiovascular diseases, diabetes and musculoskeletal disorders such as osteoarthritis [1,3,4]. Obesity is also associated with an increased risk of several cancers, such as endometrial, breast, ovarian, prostate, liver, gallbladder, kidney and colon cancer [5,6,7,8,9,10,11,12,13]. The emerging link between obesity and multiple cancer types is gaining more and more relevance in recent years [14,15,16]. Specifically, the burden of cancer attributable to obesity, expressed as affected population fraction, is 11.9% in men and 13.1% in women, and indeed obesity can be considered as one of the principal preventable causes of cancer [14]. The molecular mechanisms underlying the influence of obesity on the development and progression of cancer are not yet completely defined, and their identification and characterization could highlight new mechanisms leading to increased susceptibility to cancer.

The adipogenic microenvironment is fundamental for numerous biological and pathogenic processes and especially relevant in the context of tumor biology [17]. The survival of cancer cells is critically dependent on their interactions with neighboring non-malignant cells of the tumor stroma, and the adipose tissue within the tumor microenvironment has been shown to actively contribute to tumor growth and metastasis by functioning as an endocrine organ, through the secretion of signaling molecules, and acting as an energy reservoir for embedded cancer cells [18]. The tumor microenvironment itself can also influence adipogenesis, as it has been shown that human adipose-derived stem cells obtained from the breast cancer microenvironment present impaired peroxisome proliferator-activated receptor (PPARγ) activation and a subsequent inhibition of differentiation [19,20]. Moreover, hypertrophic expansion of adipose tissue as in the context of obesity shares many features with solid tumor growth [18]. Even so, the molecular bases of the interactions between these key mediators, cancer cells and adipocytes, which create the tumor-permissive microenvironment, are not fully known [21]. To this end, transcriptional characterization of specific tissues in obese patients, focusing on both coding and non-coding genes, is of crucial relevance in highlighting new key players.

It is indeed possible to highlight how obesity strictly correlates with cancer risk, but patient stratification could provide more insights into the specific cancer risk for a selected class of patients. To this end, it is worth analyzing if the presence of a secondary comorbidity, such as type 2 diabetes (T2D), or of gender differences, could impact cancer development. The possible biological links between diabetes mellitus and cancer comprise hyperinsulinemia, hyperglycemia and fat-induced chronic inflammation, and the strongest association refers to pancreas and liver, although there are many other organs affected by carcinogenesis in diabetic patients, including the breast, endometrium, bladder and kidney [22]. The link between diabetes and cancer has been suggested to lie in the ability of glucose, when found at elevated concentrations, to change the expression of certain genes, acting at the epigenomic level [23,24]. Indeed, numerous epidemiological and pre-clinical studies have shown an implication for the insulin-like growth factor (IGF) in the development and progression of multiple cancers.

Another important point of view is the consideration of the differences between sexes observed in cancer, as the study of the biological mechanisms responsible for sex-biased differences may yield improved cancer management and the development of personalized therapeutic strategies [25,26,27]. Until today, clinical trials and studies in animal models have been gender-unbalanced [25]. Interestingly, one research project conducted in over 410,000 adults highlighted a higher risk of developing cancer in men and women with type 2 diabetes compared to people of their own sex without metabolic problems, and increased risks were noted for 11 types of cancer in men with T2D and 13 types of cancer in women with T2D, highlighting specific gender differences [28]. In men, the risk is increased for prostate cancer (86%), but also for leukemia and lymphomas, thyroid cancer, liver cancer, kidney cancer, pancreatic cancer, colorectal cancer and stomach cancer. In women, an increased risk is found for nasopharyngeal cancer and esophageal cancer, but also liver, thyroid, lung, pancreas, blood (leukemia’s and lymphomas), uterus, colorectal, breast, cervix and stomach tumors [28].

Molecular characterization of the underlying basis of obesity is crucially needed, and a specific focus should be placed on the presence of comorbidities and gender differences. In order to investigate these aspects, the aim of this pilot study is to highlight the presence of coding and non-coding oncogenes in subcutaneous adipose tissue, along with a possible prediction of their implication in multiple cancers, in the presence of a comorbidity, such as diabetes mellitus, and considering gender differences.

## 2. Results

### 2.1. Transcriptional Differences and Substantial Deregulation in Oncogenes in SAT Tissue

**Figure 1:**
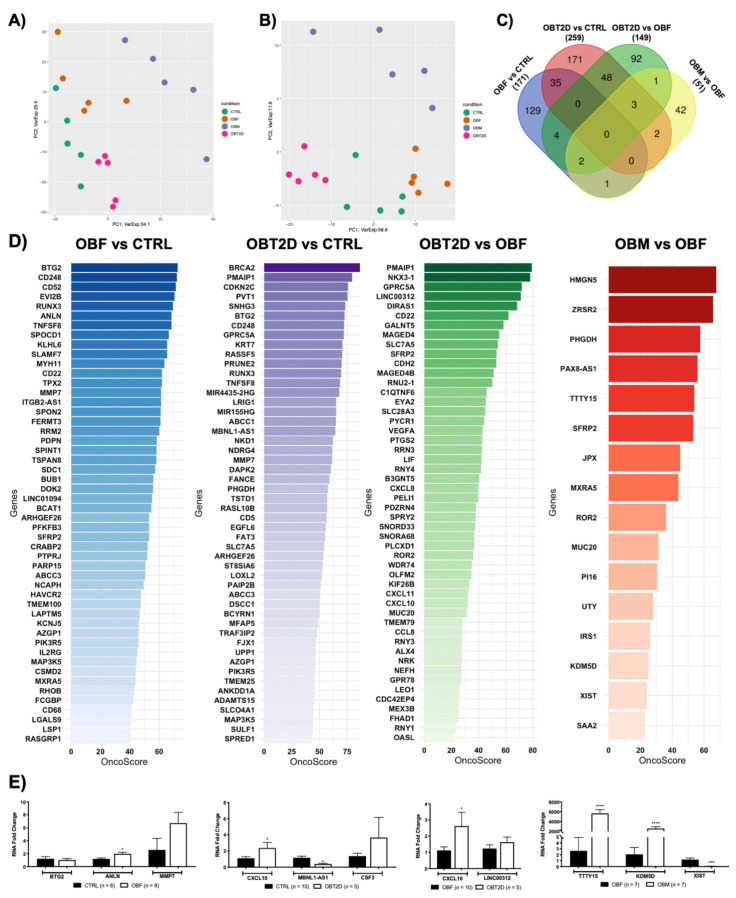
Transcriptome analysis highlights different expression profiles in SAT (Subcutaneous Adipose Tissue) of obese patients. Samples from 5 healthy, normal-weight women (CTRL), 5 obese women (OBF), 5 obese women with T2D (OBT2D) and 5 obese men (OBM) were obtained and four conditions (OBF vs. CTRL, OBT2D vs. CTRL, OBT2D vs. OBF and OBF vs. OBM) were analyzed. (**A**) Principal Component Analysis (PCA) of differentially expressed genes (DE RNAs) in the four conditions. (**B**) PCA of non-coding DE RNAs. (**C**) 171 DE RNAs were identified for OBF vs. CTRL, 259 DE RNAs were identified for OBT2D vs. CTRL, 149 DE RNAs were identified for OBT2D vs. OBF and 51 DE RNAs were identified for OBF vs. OBM. The Venn diagram displays how many genes are shared amongst conditions (http://bioinformatics.psb.ugent.be/webtools/Venn/, last accessed on 15 February 2021). (**D**) The OncoScore library was used to detect which genes, amongst the DE RNAs for each condition, were correlated with cancer. The y-axis represents the name of the DE RNAs related to cancer, the x-axis represents the OncoScore, and the color fades as the genes decrease in ranking. (**E**) mRNA expression levels were evaluated by Real-Time PCR in the different datasets for CTRL vs. OBF, CTRL vs. OBT2D, OBF vs. OBT2D and OBF vs. OBM. Data are expressed as mean ± SEM. The number of patients analyzed for each condition is reported in the figure. * *p* <0.05, **** *p* <0.0001 vs. the respective control condition.

Samples from 5 healthy, normal-weight women (CTRL), 5 obese women (OBF), 5 obese women with T2D (OBT2D) and 5 obese men (OBM) were obtained and transcriptome profiling of the four conditions (OBF vs. CTRL, OBT2D vs. CTRL, OBT2D vs. OBF and OBF vs. OBM; see Materials and Methods section for details) resulted in different expression profiles, as shown by PCA analysis, and the same can be concluded when looking at heatmap representation of the four categories (Figure 1A). A similar analysis was performed on non-coding, differentially expressed transcripts (nc-DE RNAs), showing substantial differences in this subclass (Figure 1B). Specifically, 171 DE RNAs were identified for OBF vs. CTRL, 259 DE RNAs were identified for OBT2D vs. CTRL, 149 DE RNAs were identified for OBT2D vs. OBF and 51 DE RNAs were identified for OBF vs. OBM (Figure 1C). Interestingly, several DE RNAs are commonly altered in more than one condition, although none in all four. Specifically, the 35 DE RNAs were commonly deregulated in OBF vs. CTRL and OBT2D vs. CTRL, suggesting that these are intrinsically deregulated by obesity and possibly not substantially influenced by the development of the diabetic complication. Moreover, 48 DE RNAs are shared when OBT2D is compared either to CTRL or OBF, suggesting that these could be the main driving forces in the development of T2D (Figure 1C).

The OncoScore library was used to detect which genes, amongst the DE RNAs for each condition, have been correlated with cancer [29]. To do so, the default 21.09 was set as the threshold cut-off for classification as “oncogenes” for the DE RNAs, as defined by the algorithm developers [29], and the full list of the genes and respective scores is reported in Table S1. Specifically, the percentage of oncogenes in each dataset was 66.7% (114/171) for OBF vs. CTRL, 48.6% (126/259) for OBT2D vs. CTRL, 33.6% (50/149) for OBT2D vs. OBF and 31.3 (16/51) for OBM vs. OBF. Figure 1D shows the top 50 oncogenes for OBF vs. CTRL (blue), for OBT2D vs. CTRL (purple), for OBT2D vs. OBF (greens) and all the oncogenes emerging for OBM vs. OBF (red). The expression of a panel of genes, with a relevant OncoScore and FC, was analyzed via Real-Time PCR in an independent cohort of SAT samples obtained from normal-weight females, females affected by obesity, females affected by obesity and type 2 diabetes and males affected by obesity in order to support the validity of the RNA-seq analysis (Figure 1E).

**Figure 2:**
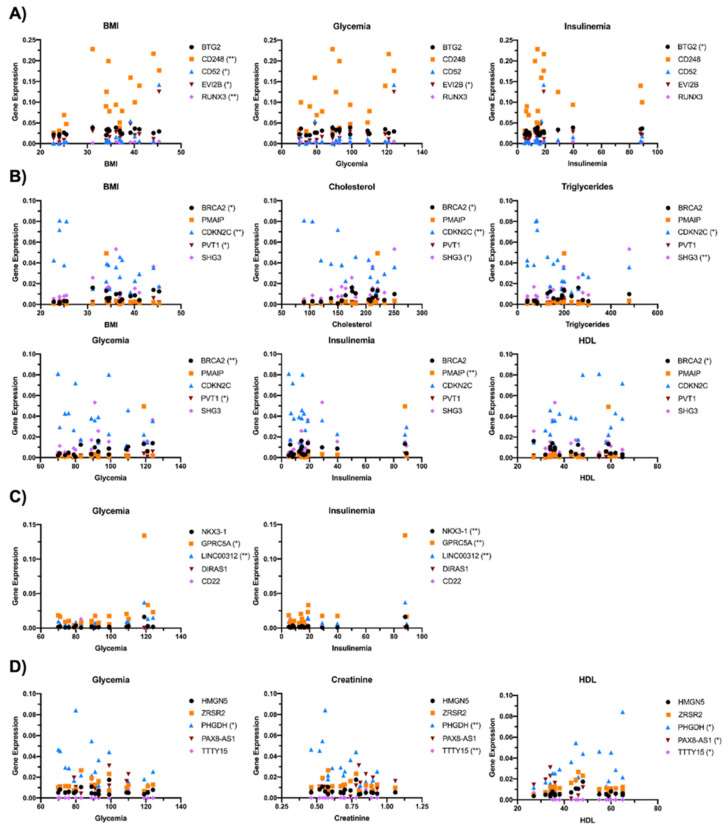
Correlation between the top 5 genes with the highest OncoScore and the subject’s anthropometrical parameters. The top 5 ranking oncogenes were correlated with Body Mass Index (BMI), cholesterol, triglycerides, glycemia, insulinemia, creatinine and High-Density Lipoproteins (HDL) (**A**) for obese women (OBF) vs. healthy controls (CTRL) (* *p* < 0.05; ** *p* < 0.01 vs. CTRL), (**B**) for obese women with type 2 diabetes (OBT2D) vs. CTRL (* *p* < 0.05; ** *p* < 0.01 vs. CTRL), (**C**) for OBT2D vs. OBF (* *p* < 0.05; ** *p* < 0.01 vs. OBF) and (**D**) for obese males (OBM) vs. OBF (* *p* < 0.05; ** *p* < 0.01 vs. OBF).

The correlation between the expression of the top five ranking oncogenes and the anthropometrical parameters (BMI, cholesterol, triglycerides, glycemia, insulinemia, creatinine and HDL) of enrolled subjects was then investigated (Figure 2). Specifically, for OBF vs. CTRL, BMI was significantly correlated with the expression of CD248, CD52, EVI2B and RUNX3, glycemia with EVI2B and insulinemia with BTG2 (Figure 2A). For OBT2D vs. CTRL, BRCA2, CDKN2C and PVT1 correlated with BMI, BRCA2, CDKN2C and SHG3 with cholesterol levels, CDKN2C and SHG3 with triglycerides, BRCA2 and PVT2 with glycemia, PMAIP with insulinemia and BRCA2 with HDL (Figure 2B). For oncogenes related to OBT2D vs. OBF, a correlation was found for GPRC5A and LINC00312 with glycemia and NKX3-1, GPRC5A and LINC00312 with insulinemia, as these are parameters which are significantly different in diabetes (Figure 2C). Lastly, DE RNAs with the top five OncoScores for OBM vs. OBF were significantly correlated with glycemia (PHGDH), creatinine (PHGDH and TTTy15) and HDL (PHGDH, PAX8-AS1 and TTTY15) (Figure 2D).

### 2.2. Both Coding and Non-Coding DE RNAs Are Associated with Cancer in Obese Subjects

**Figure 3:**
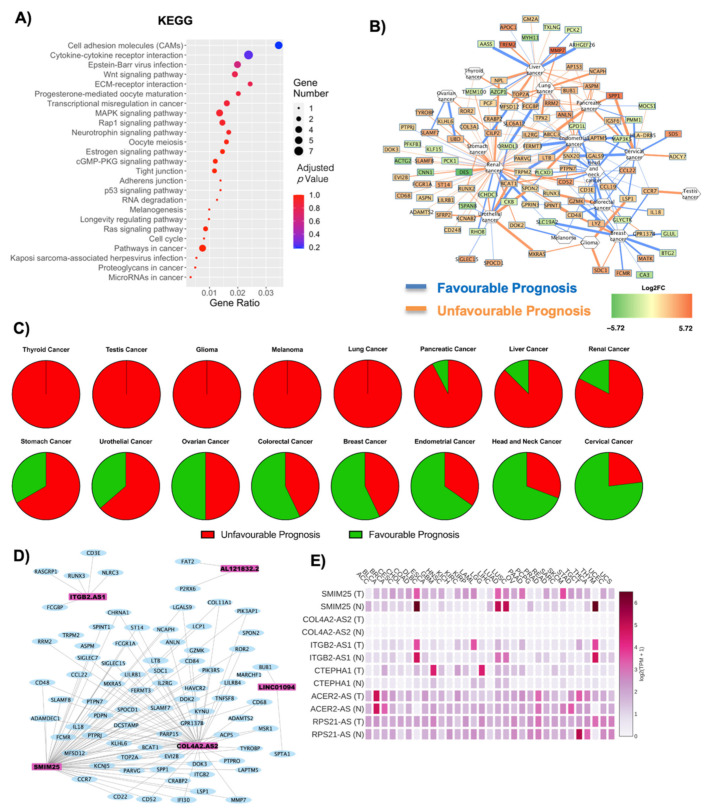
Cancer and oncogene correlations in OBF vs. CTRL conditions. (**A**) Dotplot of deregulated oncogenic pathways from KEGG analysis. The y-axis represents the name of the pathway, the x-axis represents the gene ratio, dot size represents the number of different genes and the color indicates the adjusted *p*-value. (**B**) Relationship between DE RNAs and the possibility of a cancer diagnosis. Nodes are DE RNAs and are ranked according to fold change whereas edges indicate disease prognosis and are colored according to favorable (light blue) and unfavorable (orange) prognosis. (**C**) Pie graph displays the overall unfavorable or favorable prognosis. (**D**) Co-interaction network between lncRNAs in OBF vs. CTRL and the oncogenes highlighted after OncoScore analysis. Light blue nodes are coding genes whereas pink nodes are lncRNAs. The coding and non-coding RNAs form 4 main networks of interaction, the largest of which includes both COL4A2-AS2 and SMIM25. On the contrary, ITGB2-AS1, LINC0194 (CTEPHA1) and AL121832.2 (RPS21-AS) formed each one separate interaction network. (**E**) The GEPIA2 database displays the specific annotated expression of each lncRNA in tumoral and normal tissues.

The deregulated transcripts for OBF vs. CTRL with a deregulation ≥1 in terms of |Log2FC| were subjected to pathway analysis via the enrichR web tool [30]. The oncogenic-related pathways found in the KEGG (Figure 3A) and WikiPathways (Figure S1A) analyses were highlighted and displayed as a dotplot, ranked for their significance. Specifically, 25 out of the 171 KEGG deregulated terms (15%) and 31 out of the 158 WikiPathways deregulated terms (20%) were correlated with the oncogenesis, suggesting an implication for the DE RNAs dataset in possible carcinogenesis insurgence. Interesting pathways concerning KEGG analysis that emerge specifically concern deregulation in cell adhesion molecules and cytokine–cytokine receptor interactions, transcriptional misregulation in cancer and microRNAs in cancer (indicating that the DE RNAs affect gene expression pertaining to oncogenesis), the p53 signaling pathway and even the general denomination of pathways in cancer (Figure 3A). The top deregulated pathway from KEGG analysis was the cell adhesion molecules pathway, widely implicated in cancer for its relevance in the loss of cell-to-cell adhesion and anchorage-independent growth [31,32,33]. All the DE RNAs implicated in this process were upregulated, and the represented genes codify for the MHC-II, ITGB2, CD22 and SDC proteins (Figure 3A, Figure S1B). Figure 3B reports a correlation network where the edges are correlated with disease prognosis and, specifically, for each tumor, the numbers of upregulated genes with an unfavorable prognosis (orange edges) or downregulated favorable prognosis (blue edges) were associated with an overall unfavorable prognosis, whereas downregulated genes with an unfavorable prognosis and upregulated genes with a favorable prognosis were correlated with an overall favorable prognosis (Table 1). The overall unfavorable or favorable prognosis is summarized in Figure 3C, which shows how thyroid cancer, testis cancer, glioma, melanoma and lung cancer are associated with a fully unfavorable prognosis, pancreatic cancer, liver cancer, renal cancer, stomach cancer, urothelial cancer with a predominantly unfavorable prognosis, no relevant difference for ovarian cancer and, lastly, interestingly, a predominantly favorable prognosis for colorectal cancer, breast cancer, endometrial cancer, head and neck cancer and cervical cancer (Figure 3C).

As the non-coding component is becoming increasingly relevant in the context of both obesity and cancer, closer attention was given to the implication of the six lncRNAs (SMIM25, ITGB2-AS1, COL4A2-AS2, CTEPHA1, ACER2-AS, RPS21-AS) that were found deregulated in OBF versus CTRL. Specifically, the lncRNAs’ interaction with the oncogenes highlighted after OncoScore analysis (Table S1, Figure 1D) was visualized as a WCGNA co-interaction network, to investigate whether the lncRNAs could specifically target these oncogenes (Figure 3D). The coding and non-coding RNAs form four main networks of interaction, the largest of which includes both COL4A2-AS2 and SMIM25 lncRNAs. On the contrary, ITGB2-AS1, LINC0194 (CTEPHA1) and AL121832.2 (RPS21-AS) each formed one separate interaction network (Figure 3D). The GEPIA2 database was used to obtain the specific annotated expression of each lncRNA in tumoral and normal tissues. SMIM25, followed by CTEPHA1, seems to be the one with the most significant deregulation in tumor tissues compared to normal ones, whereas COL4A2-AS2 seems to be the least implicated (Figure 3D). The specific correlation in each sample analyzed (both tumoral and normal) is reported in Figure S2, and this deregulation is summarized in Table 2 and Figure 3E. 

### 2.3. Correlation of Transcriptional Deregulation with Cancer in Obese Subjects with Type 2 Diabetes

**Figure 4:**
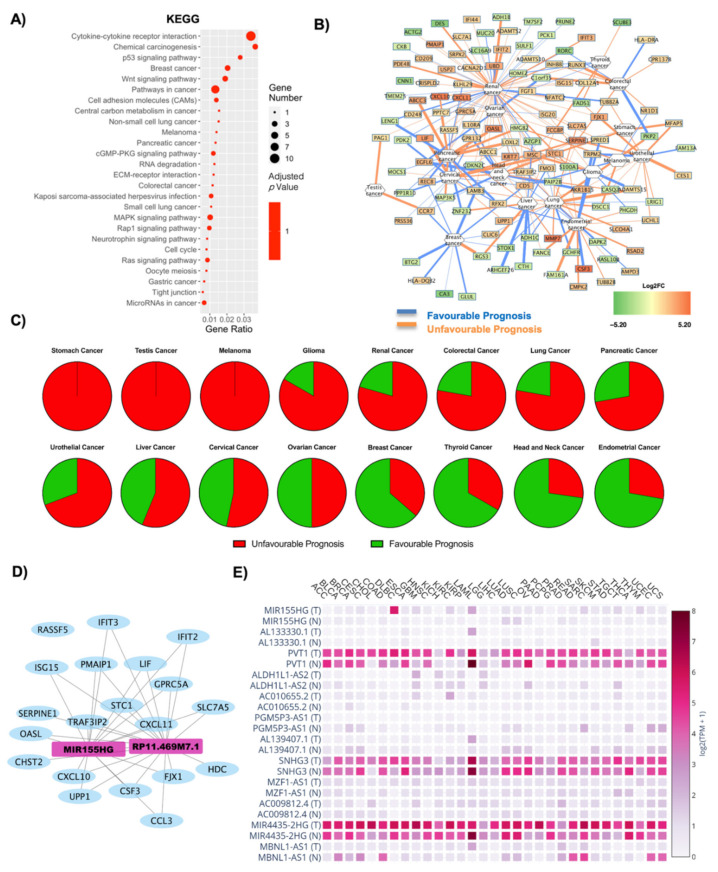
Cancer pathways and oncogene analysis in OBT2D vs. CTRL condition. (**A**) Dotplot of deregulated oncogenic pathways from KEGG analysis. The y-axis represents the name of the pathway, the x-axis represents the gene ratio, dot size represents the number of different genes and the color indicates the adjusted *p*-value. (**B**) Correlation network highlights the relationship between DE RNAs and the possibility of a cancer diagnosis. Nodes are DE RNAs and are ranked according to fold change whereas edges indicate disease prognosis and are colored according to favorable (light blue) and unfavorable (orange) prognosis. (**C**) Pie graph displays the overall unfavorable or favorable prognosis. (**D**) Co-interaction network between lncRNAs on OBT2D vs. CTRL and the oncogenes highlighted after OncoScore analysis. Light blue nodes are coding genes whereas pink nodes are lncRNAs. (**D**) The GEPIA2 database displays the specific annotated expression of each lncRNA in tumoral and normal tissues.

The deregulated transcripts for OBT2D vs. CTRL with a deregulation ≥1 in terms of |Log2FC| were subjected to pathway analysis via the enrichR web tool [30]. The oncogenic-related pathways found in the KEGG (Figure 4A) and WikiPathways (Figure S3A) analyses were highlighted and displayed as a dotplot, ranked for their significance. Specifically, 26 out of the 170 KEGG deregulated terms (15%) and 42 out of the 183 WikiPathways deregulated terms (23%) were correlated with oncogenesis phenomenon and diseases. Moreover, in this case, the KEGG analysis highlights an influence on the regulation of gene expression, with RNA degradation and microRNAs in cancer being amongst the deregulated processes, along with membrane alteration (cytokine–cytokine receptor interaction, cell adhesion molecules and ECM–receptor interaction) and implication for specific cancers (breast cancer, non-small cell lung cancer, melanoma, pancreatic cancer, colorectal cancer) (Figure 4A). The top KEGG deregulated pathway was that of cytokine–cytokine receptor interaction, also widely implicated in oncogenic processes [53,54] (Figure 4A, Figure S3B). Most of the DE RNAs implicated in this process were found to be upregulated, and they codify for CCL3L1, CCR7, CCL3, CXCL10, CSF3, LIF, IL10RA, CD30L and INHBB. The only downregulated gene found was BMP3, though, interestingly, this gene has been found downregulated in cancers [55,56]. Figure 4B reports a correlation network where the edges are correlated with disease prognosis. Moreover, in this case, there seems to be a high number of genes with unfavorable prognosis correlated with renal cancer, whist there seems to be reduced expression of genes correlating with a favorable prognosis for breast cancer. A specific analysis was performed for each tumor, and the results are reported in Table 3. The overall unfavorable or favorable prognosis is summarized in Figure 4C, which shows how obesity gene profiles are associated with a fully unfavorable prognosis in stomach cancer, testis cancer, and melanoma, glioma, renal cancer, colorectal cancer, lung cancer, pancreatic cancer, urothelial cancer, liver cancer and cervical cancer. There was no relevant difference for ovarian cancer and, lastly, a predominantly favorable prognosis for breast cancer, thyroid cancer, head and neck cancer and endometrial cancer.

The lncRNAs found in OBT2D versus CTRL were 13, and their interaction with the oncogenes (Table S1) was analyzed as a WCGNA co-interaction network, to investigate whether the lncRNAs could specifically target these oncogenes (Figure 4D). Specifically, two lncRNAs emerge to interact with the oncogenes highlighted after OncoScore analysis in one main network of interaction, and these are MIR155HG and RPM11-469M71.1 (Figure 4D). Moreover, 12 lncRNAs were found after the GEPIA2 database search (Figure 4E, Figure S4). PVT1, SNHG3 and MIR4435-2HG were amongst the most expressed terms (both in normal and tumor tissues) and the significant deregulations are reported in Table 4, and the ones with the most significant alterations were PVT1 and AL139407.1 (Table 4, Figure S4).

### 2.4. Influence of T2D on Oncogene Expression: Role of Both Coding and Non-Coding RNAs in OBT2D vs. OBF

**Figure 5:**
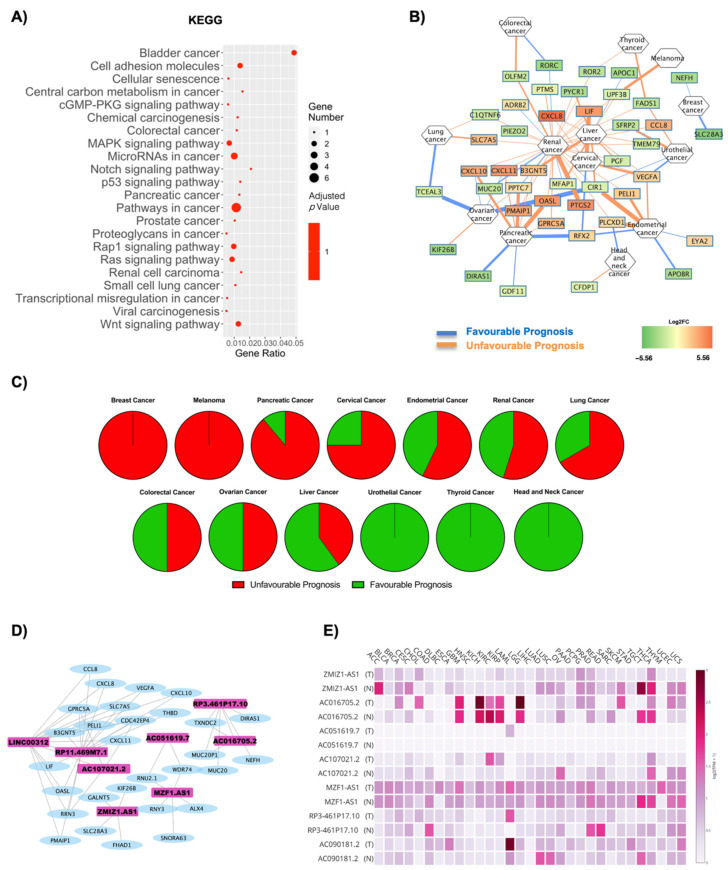
Cancer pathways and oncogene analysis in OBT2D vs. OBF. (**A**) Dotplot of deregulated oncogenic pathways from KEGG analysis. The y-axis represents the name of the pathway, the x-axis represents the gene ratio, dot size represents the number of different genes and the color indicates the adjusted p-value. (**B**) Nodes are DE RNAs and are ranked according to fold change whereas edges indicate disease prognosis and are colored according to favorable (light blue) and unfavorable (orange) prognosis. (**C**) Pie graph displays the overall unfavorable or favorable prognosis. (**D**) Co-interaction network between lncRNAs on OBT2D vs. OBF and the oncogenes highlighted after OncoScore analysis. Four networks were built, including a total of 8 lncRNAs. (**E**) The GEPIA2 database displays the specific annotated expression of each lncRNA in tumoral and normal tissues.

The deregulated transcripts for OBT2D vs. OBF with a deregulation ≥1 in terms of |Log2FC| were subjected to pathway analysis via the enrichR web tool [30]. The oncogenic-related pathways found in the KEGG (Figure 5A) and WikiPathways (Figure S5A) analyses were highlighted and displayed as a dotplot, ranked for their significance. Specifically, 22 out of the 101 KEGG deregulated terms (21.8%) and 31 out of the 120 WikiPathways deregulated terms (25.8%) were identified as correlated with oncogenesis. KEGG analysis implicates specific cancers (bladder cancer, colorectal cancer, pancreatic cancer, prostate cancer and more), along with canonical pathways such as the MAPK signaling pathway, Notch p53 signaling pathway, Wnt signaling pathway and, again, transcriptional misregulation in cancer (Figure 5A). Bladder cancer was the most deregulated pathway, previously reported to be associated with diabetes, although no molecular signature was identified for this correlation [72,73] and the upregulated genes involved in these pathways were VEGF and IL-8 (Figure 5A, Figure S5B).

Figure 5B reports a correlation network where the edges are correlated with disease prognosis. The analysis highlights an increased susceptibility for renal cancer when switching to a diabetic phenotype, although, overall, a reduced number of genes and tumors was found in the network. The specific gene–prognosis correlation is reported in Table 5, and Figure 5C summarizes for which cancers the DE RNAs signature is favorable or unfavorable. Indeed, Figure 5C shows how breast cancer and melanoma are associated with a fully unfavorable prognosis, pancreatic cancer, cervical cancer, endometrial cancer, renal cancer and lung cancer with a predominantly unfavorable prognosis, no relevant difference for colorectal and ovarian cancer, a predominantly favorable prognosis for liver cancer and, remarkably, a fully favorable prognosis for urothelial cancer, thyroid cancer and head and neck cancer.

The lncRNAs found in OBT2D versus OBF were nine, and a co-interaction network with the oncogenes was constructed (Figure 5D and Table S1). Four networks were built, including a total of eight lncRNAs. Specifically, LINC00312, RPM11-469M71.1 and AC107021.2 interact in the most complex network, AC051619.7 co-interacts with MZF1-AS1 in a smaller one, conversely to RP3-461P17.10 and AC016705.2. ZMIZ1-AS1 forms a separate independent network (Figure 5D). Moreover, seven lncRNAs were found to be expressed in cancer tissues after the GEPIA2 database search (Figure 5E, Figure S6). In this case, AC016705.2 and RP3-461P17.10 presented the highest deregulation amongst cancer tissues (Figure 5E, Table 6, Figure S6).

### 2.5. Gender Differences Lead to Alteration in Oncogene Expression in Relation to Obesity

**Figure 6:**
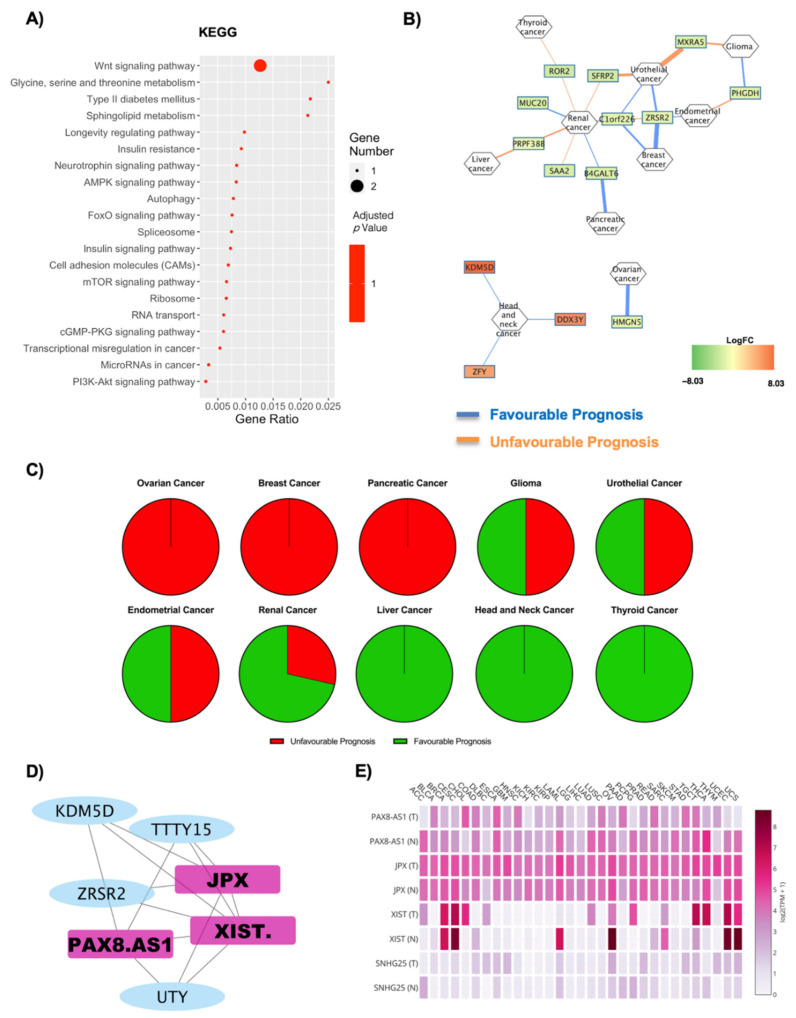
Cancer pathways and oncogene analysis concerning gender differences. (**A**) Dotplot of deregulated oncogenic pathways from KEGG analysis. The y-axis represents the name of the pathway, the x-axis represents the gene ratio, dot size represents the number of different genes and the color indicates the adjusted *p*-value. (**B**) Nodes are DE RNAs and are ranked according to fold change whereas edges indicate disease prognosis and are colored according to favorable (light blue) and unfavorable (orange) prognosis. (**C**) Pie graph displays the overall unfavorable or favorable prognosis. (**D**) Co-interaction network between lncRNAs on OBT2D vs. OBF and the oncogenes highlighted after OncoScore analysis. One main network was built, including a total of 3 lncRNAs: XIST, PAX8-AS1 and JPX. (**E**) The GEPIA2 database displays the specific annotated expression of each lncRNA in tumoral and normal tissues.

The deregulated transcripts for OBM vs. OBF with a deregulation ≥1 in terms of |Log2FC| were subjected to pathway analysis via the enrichR web tool [30]. The oncogenic-related pathways found in the KEGG (Figure 6A) and WikiPathways (Figure S7A) analyses were highlighted and displayed as a dotplot, ranked for their significance. Specifically, 20 out of the 24 KEGG deregulated terms (83.3%) and 35 out of the 43 WikiPathway deregulated terms (81.3%) were identified as correlated with oncogenesis. KEGG analysis implicates a high number of pathways correlated with gene expression regulation, such as spliceosome, ribosome, RNA transport, transcriptional misregulation in cancer and microRNAs in cancer (Figure 6A). The most significantly deregulated pathway in KEGG analysis was the Wnt signaling pathway, and the downregulated genes in this case codified for FRP and ROR1/2 (Figure 6A, Figure S7B). Figure 6B reports a correlation network where the edges are correlated with disease prognosis. Renal cancer is the one with the highest number of implicated genes, which mainly seem to be downregulated in this network. The specific gene–prognosis correlation is reported in Table 7. The overall unfavorable or favorable prognosis is summarized in Figure 6C, which shows how ovarian, breast and pancreatic cancer are associated with a fully unfavorable prognosis, while glioma, urothelial and endometrial cancer present no relevant difference. A predominantly favorable prognosis in renal cancer was found and a fully favorable prognosis for liver, head and neck and thyroid cancers, respectively (Figure 6C). The lncRNAs found in OBM versus OBF were four, and a co-interaction network with the oncogenes (Table S1) was constructed (Figure 6D). One main network was built, including a total of three lncRNAs: XIST, PAX8-AS1 and JPX (Figure 6D). Moreover, four lncRNAs were found to be expressed in cancer tissues after the GEPIA2 database search (Figure 6E, Figure S8). In this case, JPX and PAX8-AS1 present the highest expression, and, furthermore, XIST and PAX8-AS1 present the highest deregulation amongst cancer tissues (Figure 6E, Table 8, Figure S8).

## 3. Discussion

The molecular mechanisms underlying the influence of obesity on the development and progression of cancer are not yet completely defined, and their characterization could highlight new mechanisms leading to increased susceptibility to cancer. Transcriptional characterization of specific tissues in obese patients is of crucial relevance in highlighting new key players and a relevant focus should be placed on lncRNAs, as emerging evidence links them to numerous obesity-related disorders and multiple types of cancer [80,81,82,83,84,85,86,87,88,89]. In order to evaluate the role of adipose tissue gene expression in tumor development, we aimed to evaluate the presence of coding and non-coding oncogenes and cancer-associated pathways in obesity-affected subjects. To this end, the results hereby presented are a comprehensive analysis of transcriptional differences occurring in the SAT of a total of 20 subjects: 5 CTRL, 5 OBF, 5 OBT2D and 5 OBM. Four experimental conditions were analyzed: OBF vs. CTRL, OBT2D vs. CTRL, OBT2D vs. OBF and OBF vs. OBM.

The specific deregulation in each subset was then analyzed. For each dataset, the presence of genes with a high oncogenic potential was assessed, along with their correlation with anthropometrical parameters. Oncogenic-associated pathways were identified via KEGG analysis, along with the specific genes which predicted a favorable or unfavorable prognosis for a specific cancer. Lastly, specific attention was given to lncRNAs, as this new class of molecules was found to be highly implicated in both oncogenesis and adipogenesis. Indeed, all four conditions highlighted the presence of much cancer-associated evidence, predicting a plausible function for the adipose tissue in oncogene deregulation. The condition which showed the highest deregulation was that of OBF vs. CTRL, where the highest percentage of oncogenes was found (66.7%), along with the highest significance for cancer-associated pathways. Indeed, the amount of evidence correlating the adipogenic microenvironment with cancer is rising each year, and some recent studies have even found that systemic metabolisms can influence the tumor microenvironment [90]. Indeed, Ringel et al. investigated how obesity shifts the metabolic landscape of the tumor microenvironment to inhibit T cell function and promote tumor growth [90]. Although, in most cases, an excessive body weight is associated with carcinogenesis development and poor outcome, some new studies are now highlighting how this might not always be the case [67,91,92] and this is in line with our evidence highlighting a correlation with both a favorable and an unfavorable gene expression signature.

Interestingly, the diabetic condition presented a high number of oncogenic-associated lncRNAs, suggesting that these molecules could be new players in the adipogenic deregulations concerning both cancer and diabetes. Indeed, this could explain the fact that the oncogenic pathway analysis, which only considered the coding genes, presented a reduced significance. T2D pharmacotherapy could also influence this phenomenon, as recent studies suggest that there is an association between the use of anti-diabetic medications, such as metformin, a drug of choice in type 2 diabetes mellitus, and reduced cancer incidence [93]. Indeed, the protective effect of metformin was found in numerous research studies investigating breast, pancreas, liver, colon, ovaries and prostate tumors [94,95]. Moreover, evidence show how the link between diabetes and cancer seems to lie in the ability of glucose, when found at elevated concentrations, to change the expression of certain genes, acting at the epigenomic level [23,24], and this is also appreciable in our correlation studies, as in OBT2D vs. OBF, the oncogenes were correlated specifically with glycemia and insulinemia. Lastly, the gender-specific analysis allowed us to identify which cancers are more associated with a favorable or unfavorable oncogene signature, which could be sex-specific even in the adipose tissue. Indeed, recent studies highlighted how, in obese men, the risk is increased for prostate cancer, but also for leukemia and lymphomas, thyroid, liver, kidney, pancreatic, colorectal and stomach cancer, whereas in women, there is an increased risk for nasopharyngeal and esophageal cancer, and for liver, thyroid, lung, pancreas, blood (leukemia’s and lymphomas), uterus, colorectal, breast, cervix and stomach tumors [28], in line with our results.

In conclusion, our results are an extensive characterization of gene expression in SAT tissue and its correlation with cancer, in both obesity and diabetes and when considering gender differences. Our work is to be considered a pilot study, with the need for further validation in different adipose depots, wider cohorts and including follow-up studies in order to correlate the gene expression with cancer risk in obese and diabetic patients, discriminating between sexes. They could shed light on new coding and non-coding molecular targets to be specifically modulated in obesity and highlight which cancers should be given the most attention.

## 4. Materials and Methods

### 4.1. Adult Human Adipose Tissue Collection, Isolation and Differentiation

The present study is in accordance with the Declaration of Helsinki and it was approved by the Ethical Committee of IRCCS Istituto Auxologico Italiano (Ethical Committee approval code #2020_10_20_04). Signed informed consent was obtained from each enrolled patient for tissue sampling. Biopsies of SAT were collected from a total of 20 subjects: 5 healthy, normal-weight women (CTRL, age 37 ± 6.7 years, BMI 24.3 ± 0.9 kg/m^2^), 5 obese women (OBF, age 41 ± 12.5 years, BMI 38.2 ± 4.6 kg/m^2^), 5 obese women with T2D (OBT2D; age 54.6 ± 14.9 years, BMI 38.1 ± 11.8 kg/m^2^) and 5 obese men (OBM, age 42.4 ± 6.58 years, BMI 36.9 ± 3.5 kg/m^2^). The anthropometrical features of patients enrolled in the study are reported in Table S2. Surgical biopsies of whole abdominal SAT were collected pre-operatively from obese patients during bariatric surgery procedures and from normal-weight patients before aesthetic plastic surgery or abdominal surgery for non-inflammatory diseases. Each collected biopsy was weighed and stored in 1 mL of DMEM (Invitrogen Corporation, Jefferson City, MO, USA) supplemented with 2.5% bovine serum albumin (Sigma, St. Louis, MO, USA) per gram of collected tissue. The biopsy was immediately transferred to the laboratory and processed. A fragment of the whole adipose tissue biopsy was immediately frozen in liquid nitrogen for RNA extraction.

### 4.2. SAT RNA Extraction

Approximately 500 mg of frozen SAT was homogenized in RLT buffer (Qiagen, Hilden, Germany). RNA from SAT was extracted using the RNeasy Mini Kit (Qiagen, Hilden, Germany) according to the manufacturer’s protocol and samples were then treated with the RNase-Free DNase Set (Qiagen, Hilden, Germany). Concentration and quality of the extracted RNA were determined by the NanoDrop ND-1000 spectrophotometer (NanoDrop Technologies, Wilmington, DE, USA) and RNA integrity verified by gel electrophoresis.

### 4.3. Library Preparation for RNA-Seq and Bioinformatic Data Analysis

RNA-seq libraries were prepared with the CORALL Total RNA-Seq Library Prep Kit (Lexogen, Vienna, Austria) using 150 ng total RNAs from 5 healthy women, 5 obese women, 5 obese women with T2D and 5 obese men. The RiboCop rRNA Depletion Kit (Lexogen, Vienna, Austria) was used to remove rRNA. Qualities of sequencing libraries were assessed with D1000 ScreenTape Assay using the 4200 TapeStation System (Agilent, Santa Clara, CA, USA) and quantified with Qubit™ dsDNA HS Assay Kit (Invitrogen, Carlsbad, CA, USA). RNA processing was carried out using Illumina NextSeq 500 Sequencing. FastQ files were generated via llumina bcl2fastq2 (v. 2.17.1.14; https://support.illumina.com/downloads/bcl2fastq-conversion-software-v2-20.html, last accessed on 15 February 2021) starting from raw sequencing reads produced by Illumina NextSeq sequencer. Quality of individual sequences was evaluated using FastQC software (see Code Availability 1) after adapter trimming with cutadapt software. Gene and transcript intensities were computed using STAR/RSEM software [96], using Gencode Release h38 (GRCh38) as a reference, using the “-strandness forward” option. Transcript abundance was obtained using the BlueBee^®^ Genomics Platform (Lexogen, Vienna, Austria). Differential expression analysis for mRNA was performed using R package DESeq2 [97]. Genes were considered differentially expressed and retained for further analysis with |log2(condition sample/control sample) | ≥ 1 and an False Discovery Rate (FDR) ≤ 0.1. We imposed minimum |Log2FC| of 1 and an FDR lower than 0.1 as thresholds to differentially expressed genes. The raw data obtained from the RNA-seq analysis are deposited in the Gene Expression Omnibus repository with the accession number GSE166047.

### 4.4. RNA Extraction and Real-Time PCR

Real-Time PCR was performed with the StepOnePlus^TM^ Real-Time RT-PCR System (Thermo Fisher, Waltham, MA, USA) with the SsoAdvancedTM Universal SYBR ^®^ Green Supermix (Bio-Rad, Hercules, CA, USA) as dye. Primers were designed with NCBI’s Primer-BLAST tool and they are reported in Table S3. Gene expression was calculated using the 2^−ΔΔCt^ method, and 18S was used as an endogenous control. Data were expressed as mean ± SEM. The statistical analysis was performed with Student’s *t*-test. The Prism 7 software (GraphPad Software Inc., La Jolla, CA, USA) was used, assuming a *p*-value less than 0.05 as the limit of significance.

### 4.5. Pathway Analysis and Cancer Correlations

Gene enrichment analysis was performed on coding genes. Kyoto Encyclopedia of Genes and Genomes (KEGG) pathway analysis (http://www.genome.ad.jp/kegg, last accessed on 15 February 2021) and WikiPathways analysis (https://www.wikipathways.org/index.php/WikiPathways, last accessed on 15 February 2021) of differentially expressed coding genes via the enrichR web tool was performed. Moreover, Gene Ontology (GO) analysis for biological processes, cellular components and molecular function was executed [30,98]. The R software was used to generate heatmaps (heatmap.2 function from the R ggplots package), PCA plot (prcomp function from the R ggplots package), volcano plots [99], dotplot graphs (ggplot2 library) and Pathview graphs (Pathview library [100]). The NDEx plugin [101] was used to group the differentially expressed genes with their prognosis in specific cancer types, visually represented using the Cytoscape software [102]. The OncoScore library in R was used to assess the specific cancer risk for the differentially expressed RNAs, and this score was plotted using the ggplot2 library [29]. The GEPIA2 tool was used to identify lncRNA expression in cancer datasets. GEPIA is a web server composed of the RNA sequencing expression data of 9736 tumors and 8587 normal samples from the TCGA and the GTEx projects, and analysis can be performed using a standard processing pipeline [103,104].

### 4.6. Correlation Analysis

Correlation analysis was performed on the top five genes for OncoScore ranking. They were correlated with anthropometrical parameters corresponding to specific patients (Table S2). For each gene, the raw counts were normalized on the raw counts of EEF2, identified as stable housekeeping genes from the Housekeeping and Reference Transcript Atlas [105]. The Prism 8 software (GraphPad Software Inc., La Jolla, CA, USA) was used for statistical analysis, assuming a *p*-value less than 0.05 as the limit of significance.

### 4.7. Coding and ncRNA Co-Expression Analysis

Cancer-implicated coding RNAs’ co-expression with non-coding RNAs (ncRNAs) was performed using Weighted Gene Co-expression Network Analysis (WGCNA) R package (https://CRAN.R-project.org/package=WGCNA, last accessed on 15 February 2021) [106]. The soft thresholding power was chosen considering the criterion of approximate scale-free topology. Network nodes represent gene expression profiles, while undirected edge values are the pairwise correlations between gene expressions. Cytoscape software was used for network import and visualization.

## Figures and Tables

**Table 1:** Prognosis score associated with each cancer. Unfavorable or favorable prognosis was assessed according to orange or blue edges, respectively. Upregulated or downregulated genes were described according to their log2FC. A bibliographic search was performed to assess previous correlations of each cancer type with obesity.

Cancer	Unfavourable Prognosis		Favourable Prognosis		Literature Evidence for Obesity Correlation
	Upregulated Unfavourable Prognosis	Downregulated Favourable Prognosis	Downregulated Unfavourable Prognosis	Upregulated Unfavourable Prognosis	
Stomach Cancer	2	0	0	1	Negatively correlated [34,35]
Ovarian Cancer	2	0	0	2	Positively correlated [36]
Thyroid Cancer	2	0	0	0	Positively correlated [37,38]
Colorectal Cancer	2	1	0	4	Positively correlated [39,40]
Breast Cancer	2	4	0	8	Positively correlated [7,41]
Cervical Cancer	2	1	1	9	Positively correlated [8,42]
Glioma	3	1	0	0	Negatively correlated [43]
Pancreatic Cancer	10	2	1	0	Positively correlated [44,45]
Melanoma	0	0	0	2	Positive correlation amongst men [46]
Head and Neck Cancer	1	3	0	9	Positively correlated [47]
Lung Cancer	9	2	0	0	Negatively correlated [48]
Renal Cancer	44	8	4	7	Positively correlated [49]
Liver Cancer	15	6	2	1	Positively correlated [50]
Urothelial Cancer	5	2	1	3	Positively correlated [51]
Endometrial Cancer	7	1	1	14	Positively correlated [52]

**Table 2:** Correlation of DE lncRNAs with expression in cancer. Deregulation has been investigated in tumoral versus healthy tissue using GEPIA2 database and reported as ns (non-significant deregulation), a green + (upregulation in normal tissue) and a red + (upregulation in tumoral tissue). ACC: Adrenocortical carcinoma; BRCA: Breast invasive carcinoma; CESC: Cervical squamous cell carcinoma and endocervical adenocarcinoma; COAD: Colon adenocarcinoma; DLBC: Lymphoid neoplasm diffuse large B-cell lymphoma; GBM: Glioblastoma Multiforme; KIRC: Kidney renal clear cell carcinoma; KIRP: Kidney renal papillary cell carcinoma; LAML: Acute myeloid leukemia; LGG: Brain lower grade glioma; LIHC: Liver hepatocellular carcinoma; LUAD: Lung adenocarcinoma; LUSC: Lung squamous cell carcinoma; OV: Ovarian serous cystadenocarcinoma; PAAD: Pancreatic adenocarcinoma; READ: Rectum adenocarcinoma; SKCM: Skin cutaneous melanoma; STAD: Stomach adenocarcinoma; TGCT: Testicular germ cell tumors; THYM: Thymoma; UCEC: Uterine corpus endometrial carcinoma.

Gene Symbol	ACER2-AS	COL4A2-AS	CTEPHA1	ITGB2-AS1	RPS21-AS	SMIM25
Tumoral Tissue						
ACC	ns	ns	ns	ns	ns	+
BRCA	ns	ns	+	ns	ns	ns
CESC	ns	ns	ns	ns	ns	+
COAD	ns	ns	ns	ns	ns	+
DLBC	ns	ns	ns	+	+	+
GBM	ns	ns	+	ns	ns	+
KIRC	ns	ns	+	+	ns	+
KIRP	ns	ns	+	ns	ns	+
LAML	ns	ns	ns	ns	+	ns
LGG	ns	ns	+	ns	ns	ns
LUAD	ns	ns	ns	+	ns	+
LUSC	ns	ns	ns	+	ns	+
OV	ns	ns	+	ns	ns	+
PAAD	ns	ns	+	+	ns	+
READ	ns	ns	ns	ns	ns	+
SKCM	+	ns	ns	ns	ns	+
STAD	ns	ns	ns	ns	ns	+
TGCT	+	ns	ns	+	+	+
THYM	+	ns	ns	+	+	+
UCEC	ns	ns	ns	ns	ns	+

**Table 3:** Prognosis score associated with each cancer. Unfavorable or favorable prognosis was assessed according to orange or blue edges, respectively. Upregulated or downregulated genes were described according to their log2FC. A bibliographic search was performed to assess previous correlations of each cancer type with T2D.

Cancer	Unfavourable Prognosis		Favourable Prognosis		Column Name Literature Evidence
	Upregulated Unfavourable Prognosis	Downregulated Favourable Prognosis	Downregulated Unfavourable Prognosis	Upregulated Unfavourable Prognosis	
Stomach Cancer	4	0	0	0	Negatively correlated [57]
Ovarian Cancer	3	2	0	5	Positively correlated [58]
Thyroid Cancer	1	1	2	2	Negatively correlated [59,60]
Colorectal Cancer	5	2	1	1	Positively correlated [61]
Breast Cancer	0	4	1	6	Positively correlated [62,63]
Cervical Cancer	6	2	1	6	Positively correlated [64]
Glioma	4	1	0	1	Negatively correlated [65]
Pancreatic Cancer	9	4	2	3	Positively correlated [46]
Melanoma	0	0	2	2	Positively correlated [66]
Head and Neck Cancer	2	1	1	7	Positively correlated [67,68]
Lung Cancer	9	5	2	2	Negatively correlated [69]
Renal Cancer	45	13	10	5	Positively correlated [70]
Liver Cancer	5	4	6	1	Positively correlated [71]
Urothelial Cancer	8	1	3	1	Positevly correlated in men [72]
Endometrial Cancer	5	0	6	7	Positively correlated [64]

**Table 4:** Correlation of lncRNAs with expression in cancer. Deregulation has been investigated in tumoral versus healthy tissue using GEPIA2 database and reported as ns (non-significant deregulation), a green + (upregulation in normal tissue) and a red + (upregulation in tumoral tissue). ACC: Adrenocortical carcinoma; BLCA: Bladder urothelial carcinoma; BRCA: Breast invasive carcinoma; CESC: Cervical squamous cell carcinoma and endocervical adenocarcinoma; COAD: Colon adenocarcinoma; DLBC: Lymphoid neoplasm diffuse large B-cell lymphoma; ESCA: Esophageal Carcinoma; GBM: Glioblastoma Multiforme; HNSC: Head and neck squamous cell carcinoma; KICH: Kidney chromophobe; KIRC: Kidney renal clear cell carcinoma; KIRP: Kidney renal papillary cell carcinoma; LAML: Acute myeloid leukemia; LIHC: Liver hepatocellular carcinoma; LUAD: Lung adenocarcinoma; LUSC: Lung squamous cell carcinoma; OV: Ovarian serous cystadenocarcinoma; PAAD: Pancreatic adenocarcinoma; READ: Rectum adenocarcinoma; SKCM: Skin cutaneous melanoma; STAD: Stomach adenocarcinoma; TGCT: Testicular germ cell tumors; THCA: Thyroid carcinoma; THYM: Thymoma; UCEC: Uterine corpus endometrial carcinoma; UCS: Uterine carcinosarcoma.

Gene Symbol →	AC009812.4	ACO10655.2	AL133330.1	AL139407.1	ALDH1L1-AS2	MBNL1-AS1	MIR155HG	MIR4435-2HG	MZF1-AS1	PGM5P3-AS1	PVT1	SNHG3
Tumoral Tissue ↓												
ACC	ns	ns	ns	ns	ns	ns	ns	ns	ns	ns	ns	+
BLCA	ns	ns	ns	ns	ns	+	ns	+	ns	ns	ns	ns
BRCA	ns	ns	ns	+	ns	+	ns	ns	ns	+	ns	ns
CESC	ns	ns	ns	+	ns	+	ns	+	ns	+	ns	ns
COAD	ns	ns	ns	ns	ns	+	+	+	ns	ns	+	ns
DLBC	ns	ns	ns	ns	ns	ns	ns	+	+	ns	+	+
ESCA	ns	ns	ns	ns	ns	ns	ns	+	ns	ns	ns	ns
GBM	ns	+	ns	ns	ns	ns	+	+	ns	ns	+	ns
HNSC	ns	ns	ns	ns	ns	ns	ns	+	ns	ns	ns	ns
KICH	ns	ns	ns	ns	ns	ns	ns	ns	ns	ns	ns	+
KIRC	ns	+	ns	ns	ns	ns	+	+	ns	ns	+	ns
KIRP	ns	ns	ns	ns	ns	ns	ns	+	ns	ns	+	ns
LAML	+	ns	+	+	ns	+	+	+	ns	ns	+	ns
LIHC	ns	ns	ns	ns	ns	ns	ns	+	ns	ns	+	ns
LUAD	ns	ns	ns	+	ns	+	ns	ns	ns	ns	+	+
LUSC	ns	ns	ns	+	ns	ns	ns	ns	ns	+	+	+
OV	ns	ns	ns	+	+	ns	ns	+	ns	ns	+	ns
PAAD	ns	ns	ns	ns	ns	ns	ns	+	ns	ns	+	ns
READ	ns	ns	ns	ns	ns	+	ns	+	ns	ns	+	ns
SKCM	ns	ns	+	+	ns	ns	ns	+	ns	ns	ns	ns
STAD	ns	ns	ns	ns	ns	ns	ns	+	ns	ns	+	ns
TGCT	ns	ns	ns	+	+	ns	ns	+	+	ns	ns	+
THCA	ns	ns	ns	ns	ns	ns	ns	+	ns	+	+	+
THYM	+	ns	ns	ns	ns	ns	+	ns	+	ns	+	+
UCEC	ns	ns	ns	+	ns	+	ns	+	ns	+	ns	ns
UCS	ns	ns	ns	+	ns	+	ns	+	ns	+	ns	ns

**Table 5:** Prognosis score and number of deregulated genes associated with each specific cancer. Unfavorable or favorable prognosis was assessed according to orange or blue edges, respectively. Upregulated or downregulated genes were described according to their log2FC. A bibliographic search was performed to assess previous correlations of each cancer type with obesity and/or T2D.

Cancer	Unfavourable Prognosis		Favourable Prognosis		Literature Evidence
	Upregulated Unfavourable Prognosis	Downregulated Favourable Prognosis	Downregulated Unfavourable Prognosis	Upregulated Unfavourable Prognosis	
Ovarian Cancer	1	2	1	2	Positively correlated with obesity and diabetes [36,58]
Thyroid Cancer	0	0	2	0	Positively correlated in obesity [37,38]; negatively correlated in diabetes [59,60]
Colorectal Cancer	0	1	1	0	Positively correlated with obesity and diabetes [39,61]
Breast Cancer	0	2	0	0	Positively correlated with obesity and diabetes [7,41,62,63]
Cervical Cancer	3	0	0	1	Positively correlated with obesity and diabetes [8,42,64]
Pancreatic Cancer	6	2	0	1	Positively correlated with obesity and diabetes [44,45,46]
Melanoma	0	0	1	0	Positively correlated with obesity and diabetes [46,66]
Head and Neck Cancer	0	0	1	1	Positively correlated with obesity and diabetes [47,67,68]
Lung Cancer	1	1	1	0	Negatively correlated with obesity and diabetes [48,69]
Renal Cancer	14	3	12	2	Positively correlated with obesity and diabetes [49,70]
Liver Cancer	3	1	6	0	Positively correlated with obesity and diabetes [50,71]
Urothelial Cancer	0	0	2	1	Positively correlated in men both in obesity and diabetes [51,72]
Endometrial Cancer	3	1	1	2	Positively correlated with obesity and diabetes [52,64]

**Table 6:** Correlation of lncRNAs with expression in cancer. Deregulation is investigated in tumoral versus healthy tissue using GEPIA2 database and reported as ns (non-significant deregulation), a green + (upregulation in normal tissue) and a red + (upregulation in tumoral tissue). ACC: Adrenocortical carcinoma; COAD: Colon adenocarcinoma; DLBC: Lymphoid neoplasm diffuse large B-cell lymphoma; KIRC: Kidney renal clear cell carcinoma; LAML: Acute myeloid leukemia; LGG: Brain lower grade glioma; OV: Ovarian serous cystadenocarcinoma; PRAD: Prostate adenocarcinoma; READ: Rectum adenocarcinoma; SKCM: Skin cutaneous melanoma; TGCT: Testicular germ cell tumors; THYM: Thymoma.

Gene Symbol →	AC016705.2	AC051619.7	AC090181.2	AC107021.2	MZF1-AS1	RP3-461P17.10	ZMIZ1-AS1
Tumoral Tissue ↓							
ACC	ns	ns	ns	ns	ns	ns	+
COAD	ns	ns	ns	ns	ns	+	ns
DLBC	ns	ns	ns	ns	+	ns	ns
KIRC	+	ns	ns	+	ns	ns	ns
LAML	ns	ns	+	ns	ns	+	ns
LGG	+	ns	ns	ns	ns	ns	ns
OV	ns	ns	ns	+	ns	ns	ns
PRAD	ns	ns	ns	ns	ns	+	ns
READ	ns	ns	ns	ns	ns	+	ns
SKCM	+	ns	ns	ns	ns	ns	ns
TGCT	+	ns	ns	ns	+	ns	+
THYM	ns	ns	ns	ns	+	ns	ns

**Table 7:** Prognosis score associated with each cancer. Unfavorable or favorable prognosis was assessed according to orange or blue edges, respectively. Upregulated or downregulated genes were described according to their log2FC. A bibliographic search was performed to assess previous correlations of each cancer type with gender.

Cancer	Unfavourable Prognosis		Favourable Prognosis		Literature Evidence
	Upregulated Unfavourable Prognosis	Downregulated Favourable Prognosis	Downregulated Unfavourable Prognosis	Upregulated Unfavourable Prognosis	
Ovarian Cancer	0	1	0	0	Positively correlated in women [36]
Thyroid Cancer	0	0	1	0	Positively correlated in women [74]
Breast Cancer	0	2	0	0	Positively correlated both in men and in women [75]
Glioma	0	1	1	0	Positively correlated in men [43,76]
Pancreatic Cancer	0	1	0	0	Positively correlated in women [77]
Head and Neck Cancer	0	0	0	3	Negatively correlated [78]
Renal Cancer	0	2	0	5	Positively correlated in women [70]
Liver Cancer	0	0	1	0	Positively correlated in men [79]
Urothelial Cancer	0	2	2	0	Positively correlated in men [72]
Endometrial Cancer	0	1	1	0	Positively correlated in women [52]

**Table 8:** Correlation of lncRNAs with expression in cancer. Deregulation has been investigated in tumoral versus healthy tissue using GEPIA2 database and reported as ns (non-significant deregulation), a green + (upregulation in normal tissue) and a red + (upregulation in tumoral tissue). ACC: Adrenocortical carcinoma; BRCA: Breast invasive carcinoma; CESC: Cervical squamous cell carcinoma and endocervical adenocarcinoma; COAD: Colon adenocarcinoma; DLBC: Lymphoid neoplasm diffuse large B-cell lymphoma; ESCA: Esophageal Carcinoma; GBM: Glioblastoma Multiforme; KICH: Kidney chromophobe; LUAD: Lung adenocarcinoma; OV: Ovarian serous cystadenocarcinoma; PAAD: Pancreatic adenocarcinoma; READ: Rectum adenocarcinoma; SKCM: Skin cutaneous melanoma; STAD: Stomach adenocarcinoma; TGCT: Testicular germ cell tumors; THCA: Thyroid carcinoma.

Gene Symbol →	JPX	PAX8-AS1	SNHG25	XIST
Tumoral Tissue ↓				
ACC	ns	+	+	+
BRCA	ns	+	ns	ns
CESC	ns	ns	ns	+
COAD	ns	ns	ns	+
DLBC	+	ns	+	+
ESCA	ns	ns	ns	ns
GBM	+	ns	+	ns
KICH	ns	+	ns	ns
LUAD	ns	+	ns	+
OV	ns	+	ns	+
PAAD	+	+	+	ns
READ	ns	ns	ns	+
SKCM	ns	+	ns	ns
STAD	ns	ns	+	+
TGCT	+	ns	ns	+
THCA	ns	+	+	+
THYM	+	+	+	ns
UCEC	ns	+	ns	+
UCS	ns	+	ns	+
UVM	ns	ns	ns	ns

## Data Availability

The raw data obtained from the RNA-seq analysis are deposited on the Gene Expression Omnibus repository with the accession number GSE166047.

## Supplementary Materials

The following are available online at https://www.mdpi.com/1422-0067/22/4/1989/s1, Figure S1: (A) Dotplot of deregulated oncogenic pathways from WikiPathways analysis. The y-axis represents the name of the pathway, the x-axis represents the gene ratio, dot size represents the number of different genes and the color indicates the adjusted p-value. (B) Pathview of the top deregulated oncogenic pathway from KEGG analysis (cell adhesion molecules pathway). Upregulated DE RNAs are shown in red. Figure S2: GEPIA2 database displays for OBF vs. CTRL how lncRNAs are expressed in different types of cancer with respect to normal tissue. Genes more expressed in normal tissue are represented in green whereas genes more expressed in cancer tissues in red. Figure S3: (A) Dotplot of deregulated oncogenic pathways from WikiPathways analysis. The y-axis represents the name of the pathway, the x-axis represents the gene ratio, dot size represents the number of different genes and the color indicates the adjusted p-value. (B) Pathview of the top deregulated oncogenic pathway from KEGG analysis (cytokine–cytokine receptor interaction). Upregulated DE RNAs are shown in red, downregulated in green. Figure S4: GEPIA2 database displays for OBT2D vs. CTRL how lncRNAs are expressed in different types of cancer with respect to normal tissue. Genes more expressed in normal tissue are represented in green whereas genes more expressed in cancer tissues in red. Figure S5: (A) Dotplot of deregulated oncogenic pathways from WikiPathways analysis. The y-axis represents the name of the pathway, the x-axis represents the gene ratio, dot size represents the number of different genes and the color indicates the adjusted p-value. (B) Pathview of the top deregulated oncogenic pathway from KEGG analysis (bladder cancer). Upregulated DE RNAs are shown in red. Figure S6: GEPIA2 database displays for OBT2D vs. OBF how lncRNAs are expressed in different types of cancer with respect to normal tissue. Genes more expressed in normal tissue are represented in green whereas genes more expressed in cancer tissues in red. Figure S7: (A) Doplot of deregulated oncogenic pathways from WikiPathways analysis. The y-axis represents the name of the pathway, the x-axis represents the gene ratio, dot size represents the number of different genes and the color indicates the adjusted p-value. (B) Pathview of the top deregulated oncogenic pathway from KEGG analysis (Wnt signaling pathway). Downregulated DE RNAs are in green. Figure S8: GEPIA2 database displays for OBM vs. OBM how lncRNAs are expressed in different types of cancer with respect to normal tissue. Genes more expressed in normal tissue are represented in green whereas genes more expressed in cancer tissues in red. Table S1: The OncoScore library was used to detect which genes, amongst the DE RNAs for each conditions, were correlated with cancer [29]. The table reports the full list of the genes and respective score, Table S2: The anthropometrical features of patients enrolled in the study. Table S3: List of primers used in this study.

## Abbreviations

ACC	Adrenocortical carcinoma
BLCA	Bladder urothelial carcinoma
BRCA	Breast invasive carcinoma
CESC	Cervical squamous cell carcinoma and endocervical adenocarcinoma
CHOL	Cholangiocarcinoma
COAD	Colon adenocarcinoma
DLBC	Lymphoid neoplasm diffuse large B-cell lymphoma
ESCA	Esophageal Carcinoma
GBM	Glioblastoma Multiforme
HNSC	Head and neck squamous cell carcinoma
KICH	Kidney chromophobe
KIRC	Kidney renal clear cell carcinoma
KIRP	Kidney renal papillary cell carcinoma
LAML	Acute myeloid leukemia
LGG	Brain lower grade glioma
LIHC	Liver hepatocellular carcinoma
LUAD	Lung adenocarcinoma
LUSC	Lung squamous cell carcinoma
MESO	Mesothelioma
OV	Ovarian serous cystadenocarcinoma
PAAD	Pancreatic adenocarcinoma
PCPG	Pheochromocytoma and paraganglioma
PRAD	Prostate adenocarcinoma
READ	Rectum adenocarcinoma
SARC	Sarcoma
SKCM	Skin cutaneous melanoma
STAD	Stomach adenocarcinoma
TGCT	Testicular germ cell tumors
THYM	Thymoma
THCA	Thyroid carcinoma
UCS	Uterine carcinosarcoma
UCEC	Uterine corpus endometrial carcinoma
UVM	Uveal melanoma
