# Supplementary material for: Transcriptome Analysis of Subcutaneous Adipose Tissue from Severely Obese Patients Highlights Deregulation Profiles in Coding and Non-Coding Oncogenes
Source: Int J Mol Sci. 2021 Feb 17;22(4):1989. doi: 10.3390/ijms22041989 (PMC7922682; doi:10.3390/ijms22041989)
Supplement: Supplementary file 1 [file ijms-22-01989-s001.zip › Rey et al_Supplementary_Materials/SupplementaryTable_3.docx]

| 18S-FW | AGTACGCAGGGCCGGTACAGTGAAACTGCG |
| --- | --- |
| 18S-REV | CGGGTTGGTTTTGATCTGATAAATGCACGC |
| ANLN-FW | GGTGTGGTAAGTCCAGAGAGTT |
| ANLN-REV | CACCAGATTCAGCTCGAGGG |
| BTG2-FW | CACCAGATTCAGCTCGAGGG |
| BTG2-REV | TGGTGTTTGTAGTGCTCTGTGA |
| CSF3-FW | ACGCGTCTCCTGTTTTTCTG |
| CSF3-REV | TGCCTGGCCCTAAAAAAGAGTC |
| CXCL10-FW | AGTGGCATTCAAGGAGTACCT |
| CXCL10-REV | TGCAGGTACAGCGTACAGTT |
| KDM5D-FW | TGGTGTTTGTAGTGCTCTGTGA |
| KDM5D-REV | TGGTGTTTGTAGTGCTCTGTGA |
| LINC00312-FW | TCTGGCTGTTGTTGTGTTGGA |
| LINC00312-REV | GCTTATTGGCTTGGTTCGCT |
| MBNL1AS1-FW | GCAGCTGAATGAGTTGTGGC |
| MBNL1AS1-REV | ACATTTCCTGAGTGACTGCTCT |
| MMP7-FW | CGGATGGTAGCAGTCTAGGGAT |
| MMP7-REV | GGCCAAGTTCATGAGTTGCAG |
| TTTY15-FW | GGACCGGGAGATAGGAGTGT |
| TTTY15-REV | CACGGACTCCAGGTGATGAG |
| XIST-FW | TAGGTGGAGATGGGGCATGA |
| XIST-REV | GCCCAGTGGTAGTGAGCTTT |
