# Supplementary figures and images for: Transcriptome Analysis of Subcutaneous Adipose Tissue from Severely Obese Patients Highlights Deregulation Profiles in Coding and Non-Coding Oncogenes
Source: Int J Mol Sci. 2021 Feb 17;22(4):1989. doi: 10.3390/ijms22041989 (PMC7922682; doi:10.3390/ijms22041989)

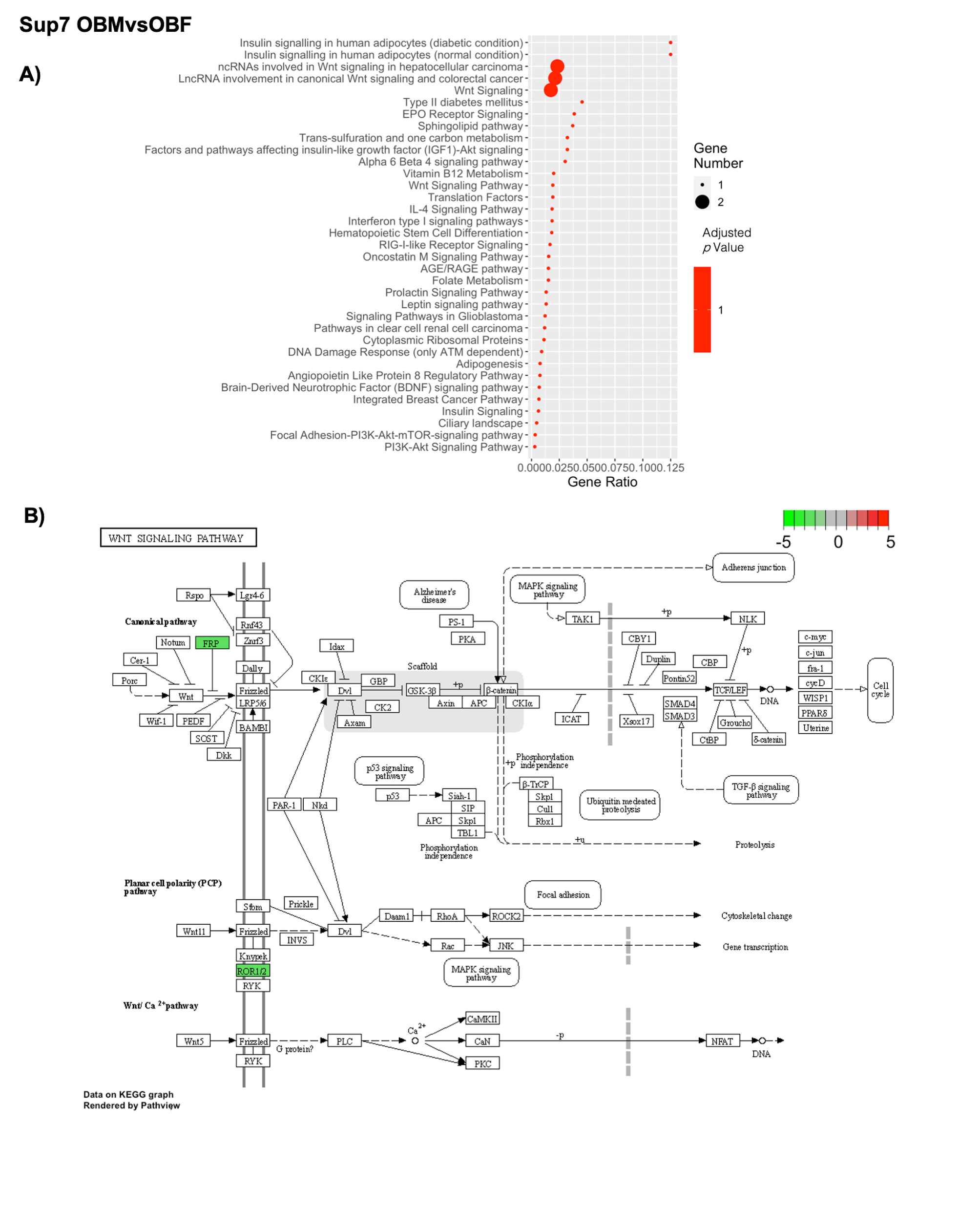

Supplement: Supplementary file 1 [file ijms-22-01989-s001.zip › Rey et al_Supplementary_Materials/FigureS7.tiff]

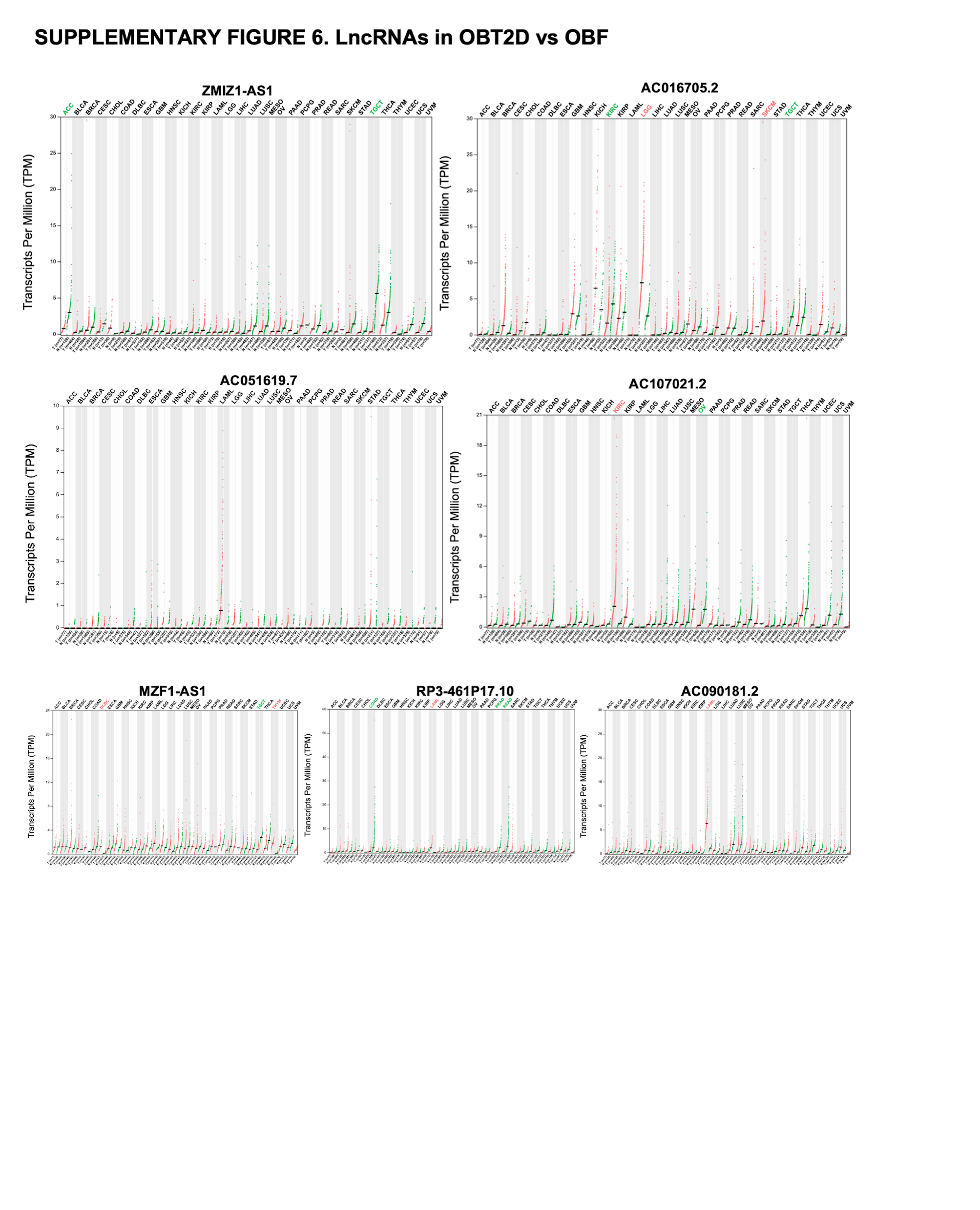

Supplement: Supplementary file 1 [file ijms-22-01989-s001.zip › Rey et al_Supplementary_Materials/FigureS6.tiff]

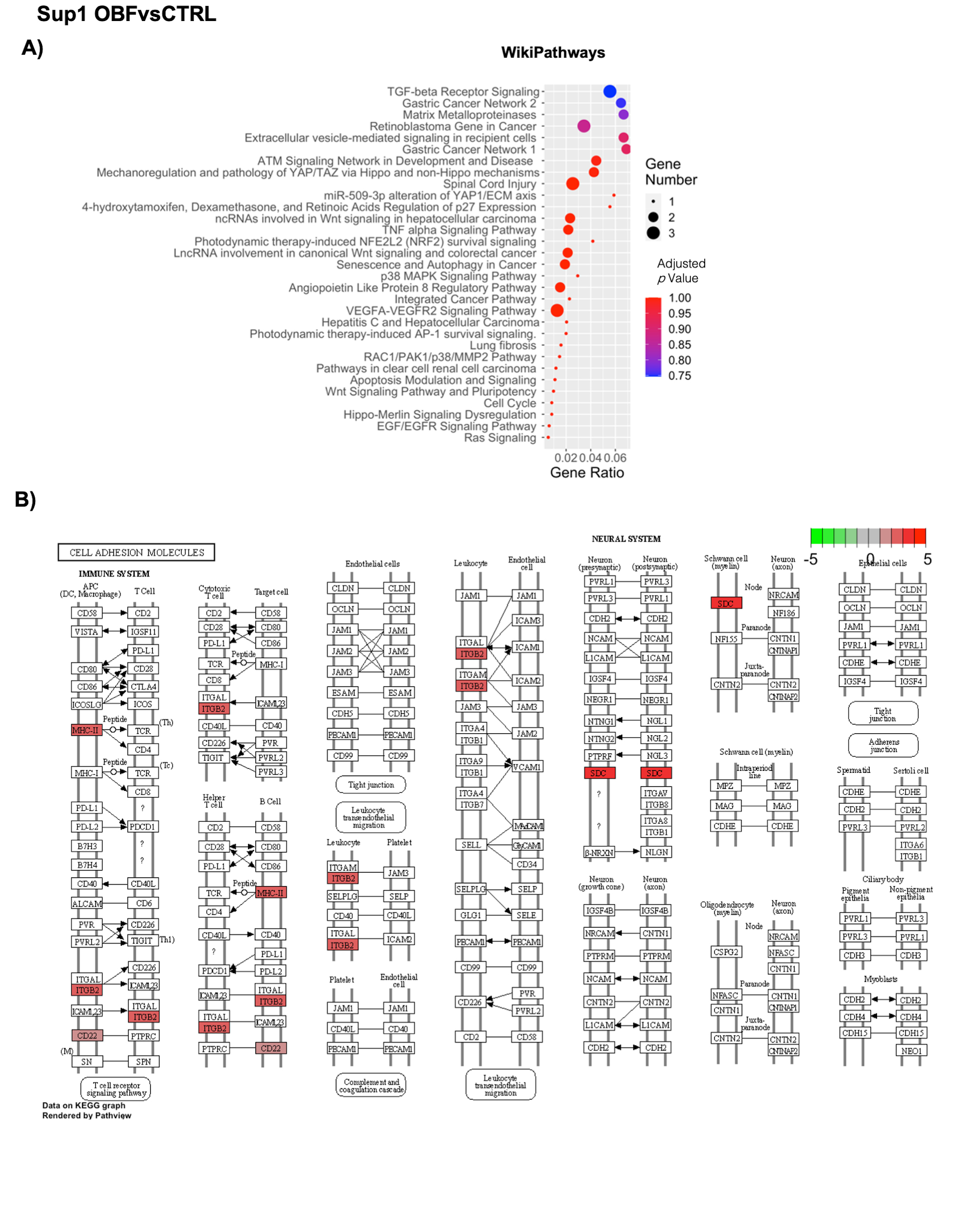

Supplement: Supplementary file 1 [file ijms-22-01989-s001.zip › Rey et al_Supplementary_Materials/FigureS1.tiff]

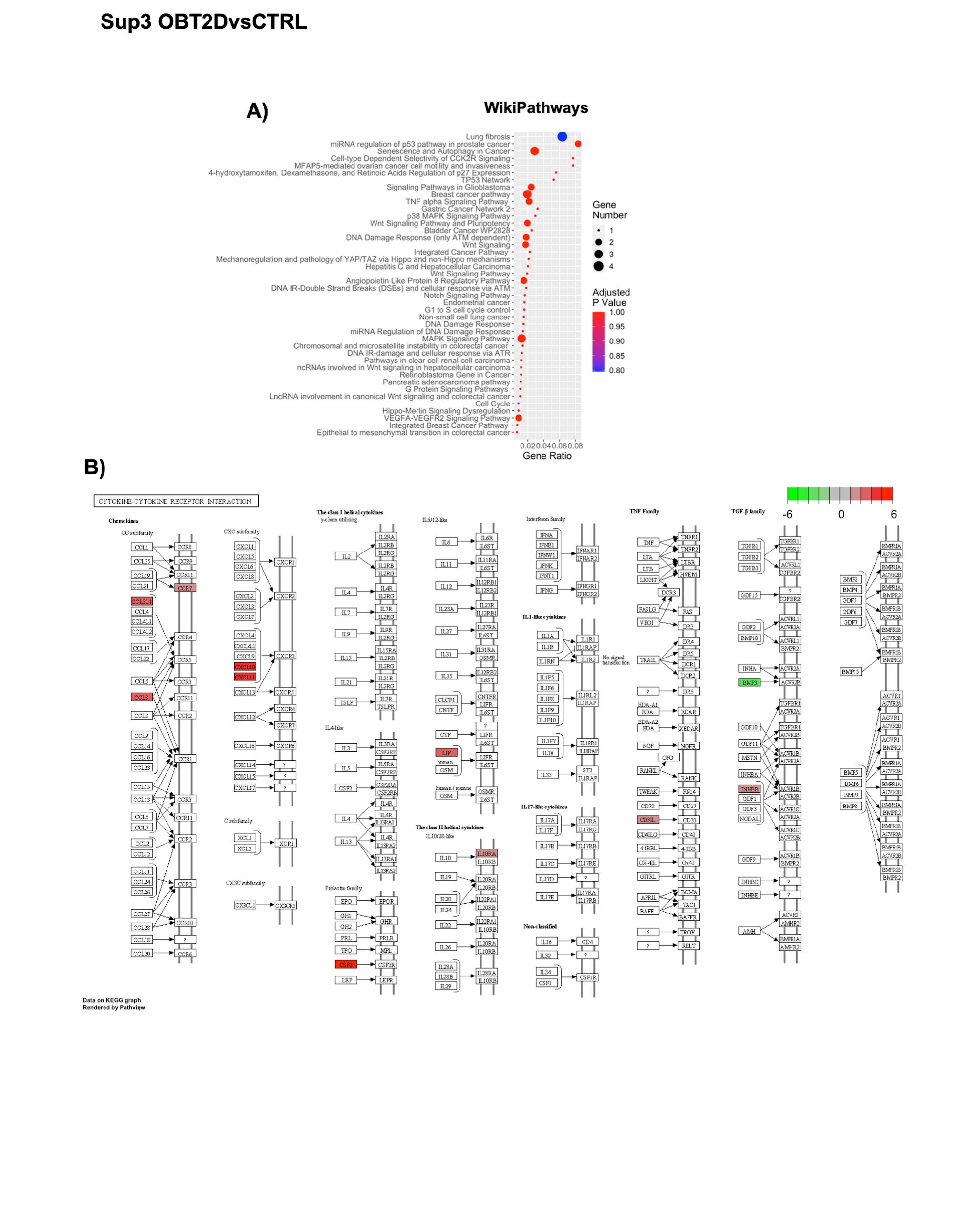

Supplement: Supplementary file 1 [file ijms-22-01989-s001.zip › Rey et al_Supplementary_Materials/FigureS3.tiff]

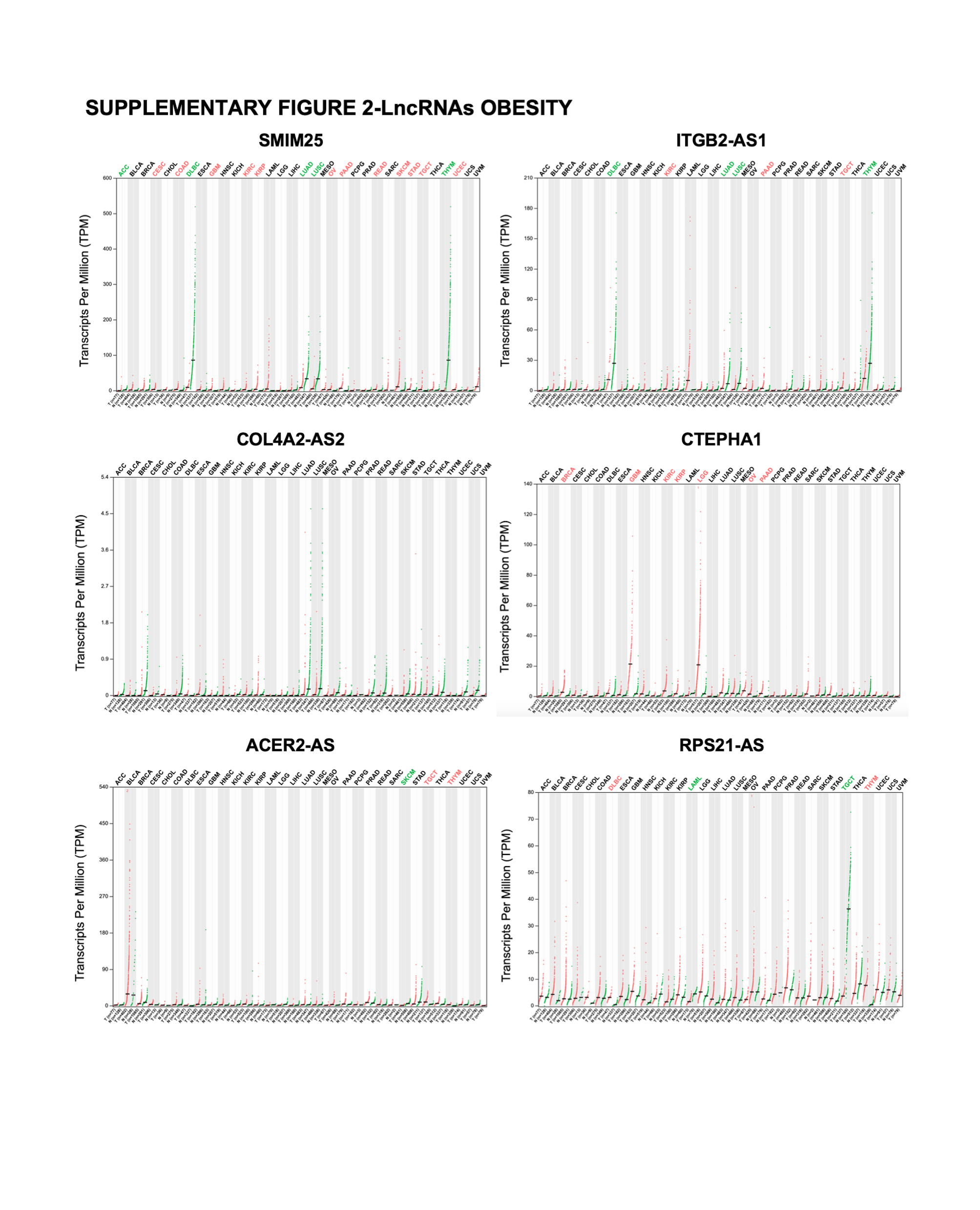

Supplement: Supplementary file 1 [file ijms-22-01989-s001.zip › Rey et al_Supplementary_Materials/FigureS2.tiff]

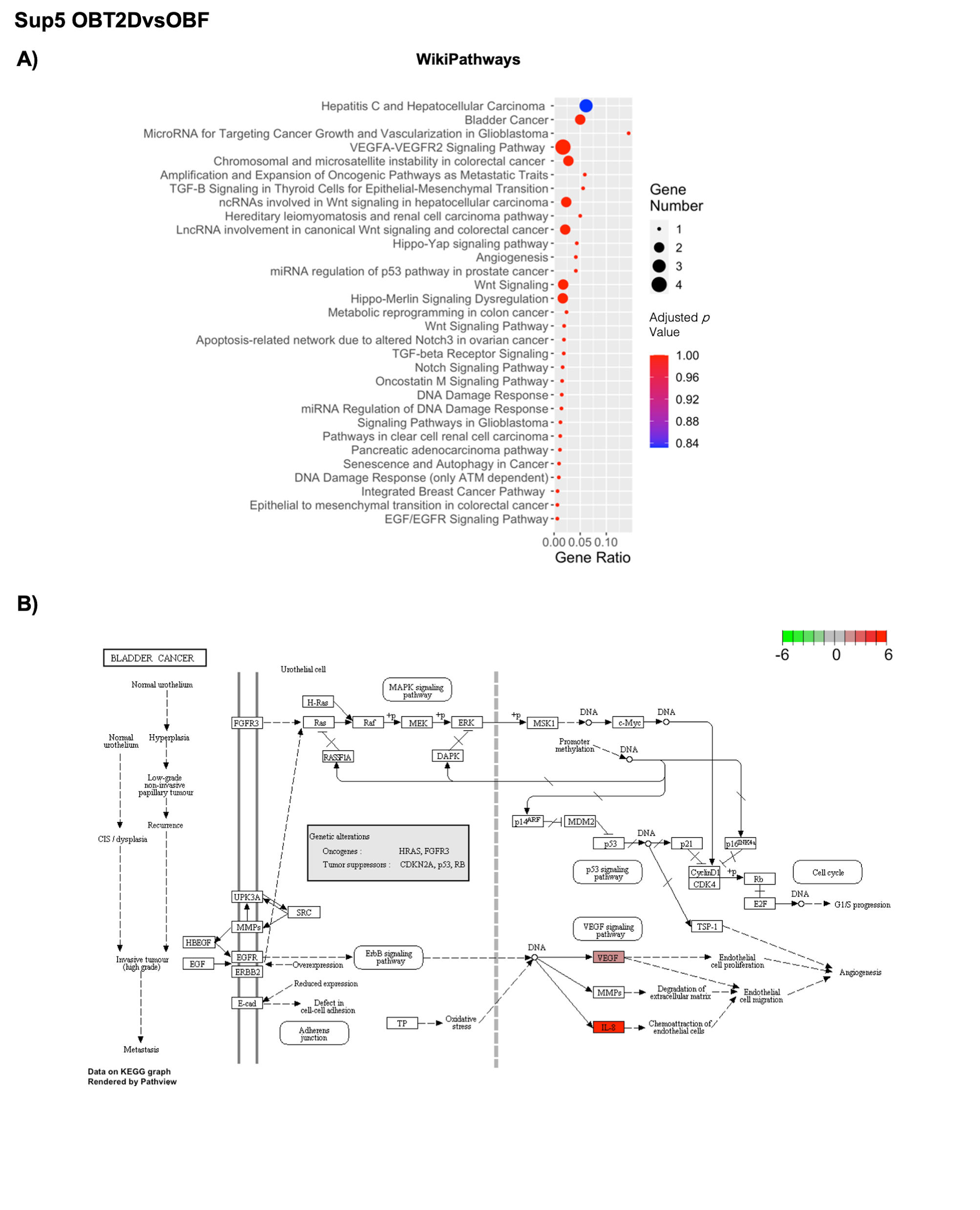

Supplement: Supplementary file 1 [file ijms-22-01989-s001.zip › Rey et al_Supplementary_Materials/FigureS5.tiff]

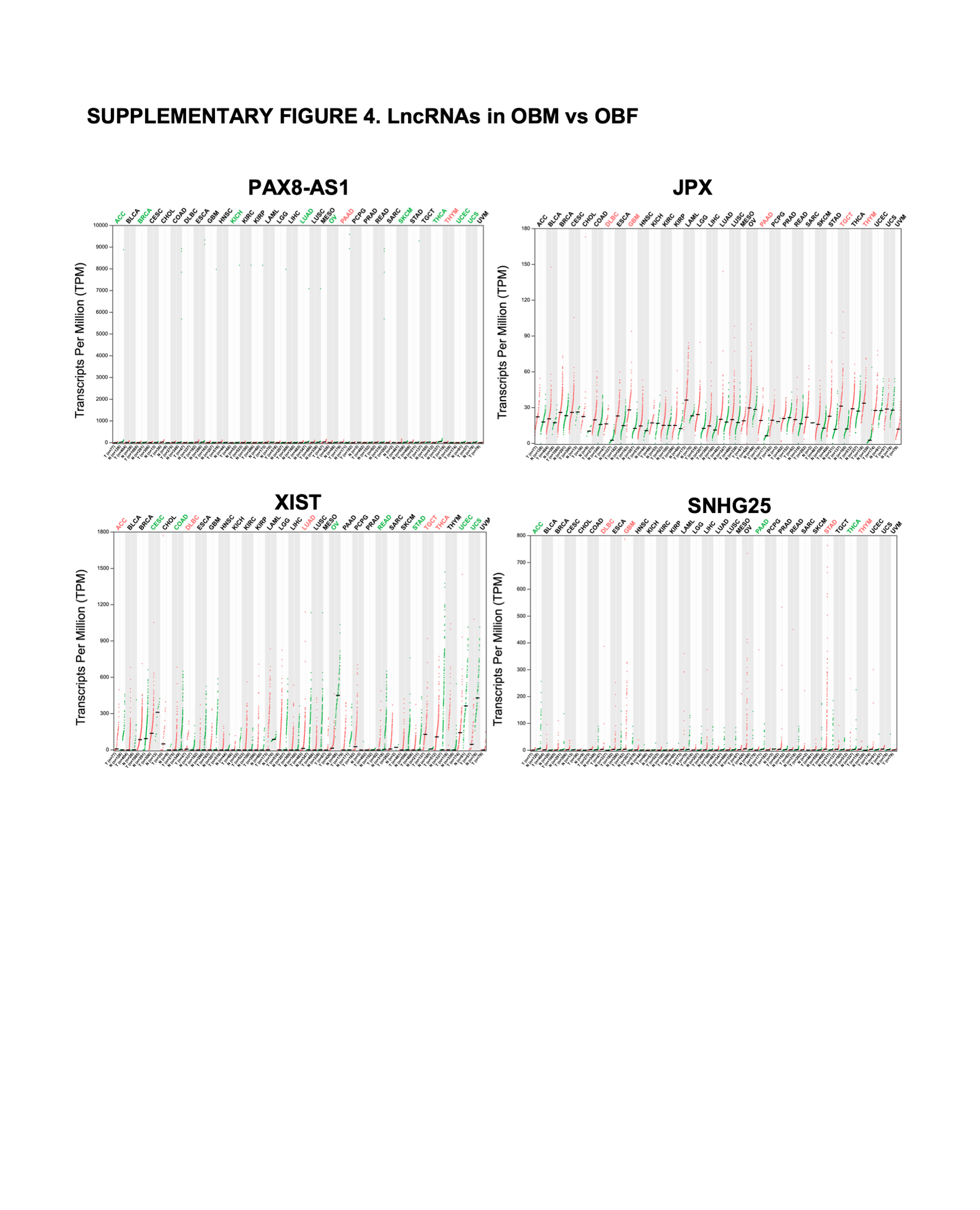

Supplement: Supplementary file 1 [file ijms-22-01989-s001.zip › Rey et al_Supplementary_Materials/FigureS8.tiff]

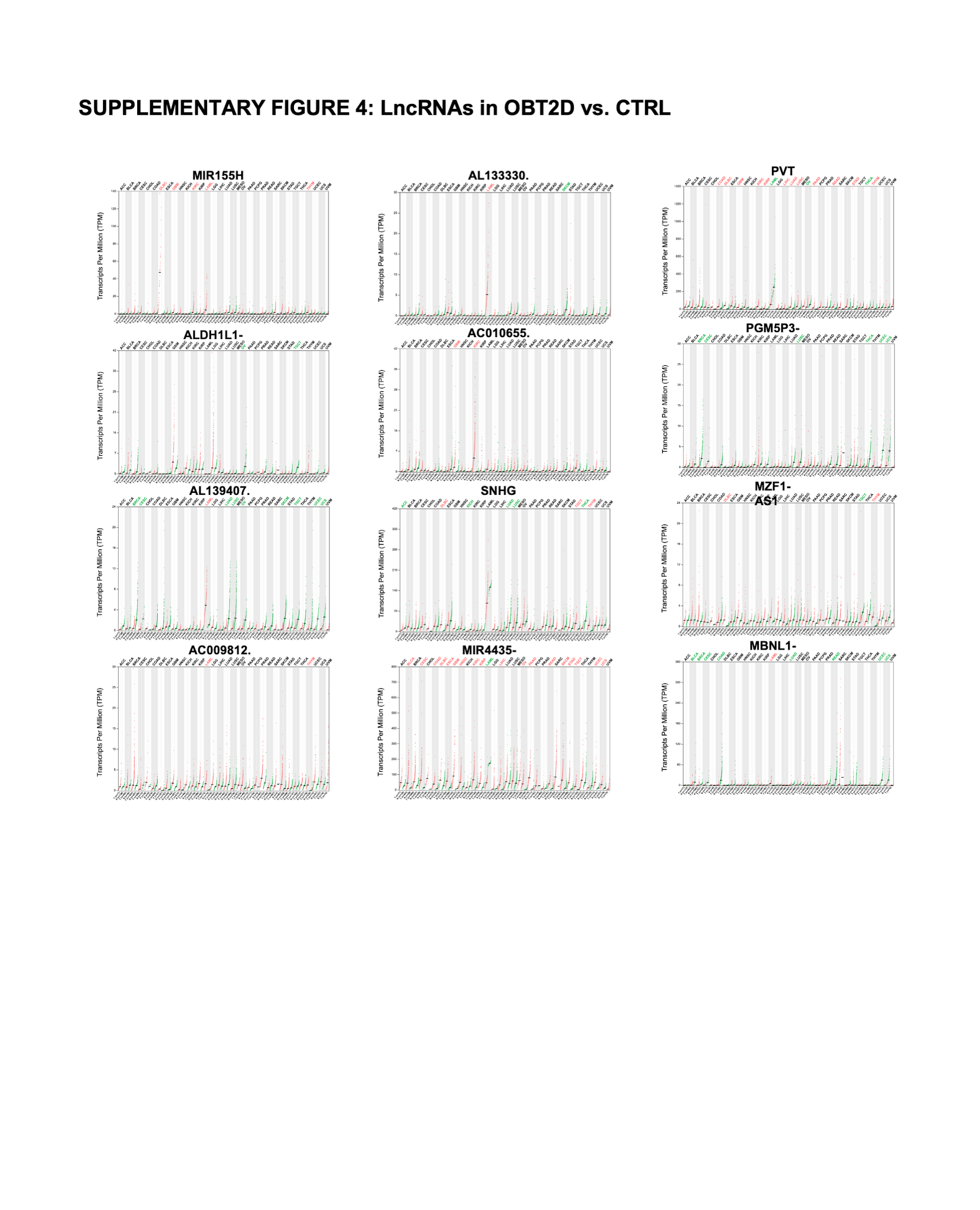

Supplement: Supplementary file 1 [file ijms-22-01989-s001.zip › Rey et al_Supplementary_Materials/FigureS4.tiff]
